# Discovery of small molecules targeting the tandem tudor domain of the epigenetic factor UHRF1 using fragment-based ligand discovery

**DOI:** 10.1038/s41598-020-80588-4

**Published:** 2021-01-13

**Authors:** Lyra Chang, James Campbell, Idris O. Raji, Shiva K. R. Guduru, Prasanna Kandel, Michelle Nguyen, Steven Liu, Kevin Tran, Navneet K. Venugopal, Bethany C. Taylor, Matthew V. Holt, Nicolas L. Young, Errol L. G. Samuel, Prashi Jain, Conrad Santini, Banumathi Sankaran, Kevin R. MacKenzie, Damian W. Young

**Affiliations:** 1 Department of Pharmacology and Chemical Biology, Baylor College of Medicine, Houston, TX 77030 USA; 2 Center for Drug Discovery, Baylor College of Medicine, Houston, TX 77030 USA; 3 Department of Chemistry, Rice University, Houston, TX 77030 USA; 4 Department of Biochemistry and Molecular Biology, Baylor College of Medicine, Houston, TX 77030 USA; 5 Department of Pathology and Immunology, Baylor College of Medicine, Houston, TX 77030 USA; 6 Lawrence Berkeley National Laboratory, Berkeley, CA 94720 USA; 7 Therapeutic Innovations Center, Baylor College of Medicine, Houston, TX 77030 USA

**Keywords:** Chemical tools, Cancer, Drug screening

## Abstract

Despite the established roles of the epigenetic factor UHRF1 in oncogenesis, no UHRF1-targeting therapeutics have been reported to date. In this study, we use fragment-based ligand discovery to identify novel scaffolds for targeting the isolated UHRF1 tandem Tudor domain (TTD), which recognizes the heterochromatin-associated histone mark H3K9me3 and supports intramolecular contacts with other regions of UHRF1. Using both binding-based and function-based screens of a ~ 2300-fragment library in parallel, we identified 2,4-lutidine as a hit for follow-up NMR and X-ray crystallography studies. Unlike previous reported ligands, 2,4-lutidine binds to two binding pockets that are in close proximity on TTD and so has the potential to be evolved into more potent inhibitors using a fragment-linking strategy. Our study provides a useful starting point for developing potent chemical probes against UHRF1.

## Introduction

**Figure 1:**
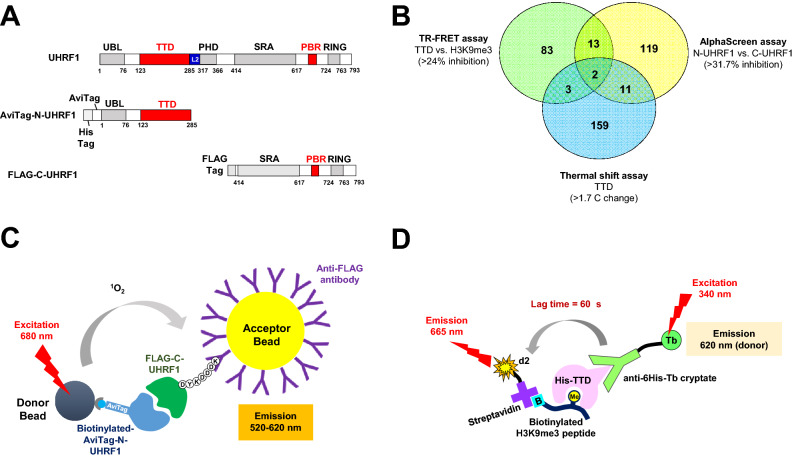
Three parallel assays identify TTD-targeting fragments by screening against the ~ 2300-compound Life Chemical fragment library (**A**) The domain structure of UHRF1 and the two truncated constructs, Avitag-N-UHRF1 and FLAG-C-UHRF1 for AlphaScreen. (**B**) The Venn diagram represents the level of hits overlaping between the three parallel screening assays. (**C**) Experimental setting for the AlphaScreen-based screening assay for fragments targeting N-UHRF1-C-UHRF1 interaction. (**D**) Experimental setting for the TR-FRET screening assay for identifying fragments that inhibit His-TTD interaction with H3K9me3 peptide.

DNA methylation at gene promoters is associated with transcriptional repression^1^ and erroneous DNA methylation marks are linked to cancer development^2^. Recruitment of the DNA methyltransferase DNMT1, which installs CpG methyl groups on the nascent (non-methylated) strand in DNA replication, is regulated by ubiquitin-like with plant and homeodomain and really interesting new gene finger domains 1 (UHRF1)^3–6^. UHRF1 acts as a macromolecular hub through five known functional domains (Fig. 1A). The UHRF1 SET- and RING-associated (SRA) domain (aa 435–586) interacts with hemi-methylated DNA^7,8^. Two epigenetic reader domains—the tandem Tudor domain (TTD; aa 126–285), which recognizes the H3K9me3 histone mark, and the plant and homeodomain (PHD; aa 310–366), which binds the non-modified Arg2 of the H3 tail (H3R2)^9–13^—direct UHRF1 to silenced nucleosomes via the H3 tail. The ubiquitin-like (UBL) domain (aa 1–76) recruits the E2 ubiquitin conjugating enzyme UBcH5a^14,15^, and the really interesting new gene (RING) E3 ligase domain (aa 724–763) directs ubiquitylation of the histone H3 subunit, which in turn recruits DNMT1^16–19^. UHRF1 is overexpressed in cancers, where it inhibits the expression of many tumor suppressor genes, including BRCA1, P16 and P21^20–25^. Given its role in cancer, UHRF1 is an attractive therapeutic target, but efforts to develop chemical modulators have met with limited success^26–31^. Some UHRF1 domains can interact with other targets, and intramolecular interactions compete with inter-molecular UHRF1 contacts^15,32–35^. As it is not clear which contacts are most crucial for UHRF1 oncogenic activity, developing chemical probes against specific UHRF1 domains could help to establish the role of each domain in promoting cancer and potentially lead to cancer therapies.

We focused on identifying binders to the UHRF1 tandem Tudor domain (TTD). Tudor domains are found in 41 human proteins^36^ and recognize specific methylated Arg or Lys residues on histones and other proteins. Tudor domains contact both the methylated residue and adjacent residues^37^ and are usually found as multiple repeats or next to other epigenetic reader domains to provide multivalency in target recognition^37^. We hypothesized that the UHRF1 TTD H3K9me3 binding pocket might be targeted by small molecules with specificity. The TTD also makes functional intramolecular interactions with the polybasic region (PBR; aa 647–678) and the linker (L2) between TTD and PHD domains (Fig. 1A) that control UHRF1 conformational states^9,10,27,32–34,38^, and it can drive high affinity interaction with DNA ligase 1 (LIG1)^39^, which joins the lagging strand Okazaki fragments that are generated during DNA replication and prevents the formation of “nicked” DNA and genetic mutations^40^. Given these important roles, compounds that target UHRF1-TTD activities could be useful chemical probes for studying UHRF1 functions and determining which domain contributes to cancer. Since these TTD functions are mediated through protein–protein interactions (PPIs), the TTD is considered a non-traditional and more challenging drug target. We implemented a fragment-based ligand discovery (FBLD) approach because of its previously documented successes in the discovery of compounds targeting epigenetic readers^41–44^.

FBLD involves screening a library of ‘fragment’ compounds that typically obey the “rule of 3”: molecular weight less than 300 Da, cLogP ≤ 3, and both the number of hydrogen bond donors and hydrogen bond acceptors ≤ 3^45,46^. Fragments contain fewer heavy atoms than molecules used in traditional high-throughput screening libraries, affording them several noteworthy advantages. The small size of fragments makes the exploration of chemical space more efficient, and in FBLD only hundreds to a few thousand fragments typically need to be screened to achieve acceptable hit rates^47^. Fragments tend to bind target proteins weakly but with high ligand efficiency (high binding free energy per heavy atom). Accordingly, fragments can serve as good starting points for ‘growing’ larger and more potent drug-like compounds. Fragments can efficiently probe the shallow binding pockets often found on PPI interfaces because they have fewer unproductive steric interactions relative to larger molecules^48^. Previous screening campaigns on the TTD domain indicate that the TTD is a druggable UHRF1 domain^26,27^. Here, we describe our efforts to interrogate the UHRF1 TTD using fragment-based screening with biophysical methods and to characterize the interaction between a selected fragment hit and TTD by protein NMR and co-crystal structure determination.

Biophysical binding experiments, which measure a physical property that changes with direct ligand:target engagement, typically serve as the basis for fragment-based screening. The high concentrations used in fragment screening often lead to false positive results, while the weak binding of fragments can produce false negatives. Biophysical binding experiments do not report on whether ligand engagement to the target modulates its intrinsic activity; while binding is a prerequisite for perturbing function, a fragment may bind without any functional effects. Function-based screens, on the other hand, provide a readout that is associated with the intrinsic biological function of a target and are hence an indirect measurement of binding. Such screens measure activity through a narrow lens, and if used alone, may miss true binders that modulate other functions. To address these limitations, we decided to pursue a multi-pronged screening strategy, using a direct biophysical binding assay to identify all fragments that bind to UHRF1-TTD in a functionally agnostic fashion, and using two function-based screens based on well-established studies of UHRF1-TTD binding partners to report on the ability of fragments to disrupt specific protein–protein interactions.

To identify binders to the UHRF1-TTD, we screened a ~ 2300-member fragment library using three parallel assays, seeking compounds that: (1) increased the melting temperature of UHRF1-TTD by a thermal shift assay (TSA) (2) perturbed the interaction between the TTD and PBR on two truncated UHRF1 proteins by AlphaScreen^49^ and (3) inhibited the TTD interaction with H3K9me3 peptide using a time-resolved fluorescence energy transfer (TR-FRET) assay. Based on the analysis of hits from all three assays, 2,4-lutidine was selected for protein NMR and co-crystallization studies with the TTD domain. The co-crystal structure obtained with 2,4-lutidine shows that binding occurs at two adjacent pockets: the aromatic cage and the Arg-binding cavity. The fragments that we identify here represent starting points toward generating probes with cellular and in vivo activities that target UHRF1. We anticipate that the experimental flow used in these studies may be further applied to discovering fragment hits against other domains of UHRF1 as well as other epigenetic proteins.

## Results and discussion

### Three parallel fragment screens to identify TTD-targeting hits

We used three different assays in parallel to screen a ~ 2300-fragment library to identify hits that bind to TTD or inhibit its activities. The first screening method was a direct binding assay, which generally serves as the basis for most fragment screening. Using the isolated His_6_-TTD (aa 123–283) we performed a thermal shift assay (TSA) to determine the change in melting temperature upon fragment binding. The change in protein melting temperature (ΔT_m_) caused by each fragment was calculated by comparison to the DMSO controls on the same plate. Using three standard deviations (> 1.7 °C increase of T_m_) as the cut off, 175 fragments (7.5% hit rate) were identified as stabilizers of His_6_-TTD (Fig. 1B).

We next turned our attention to screens that reported on functional effects of fragment binding to UHRF1-TTD. UHRF1 can assume two functionally distinct conformations that are favored by different intra-molecular interactions^15,32–35^. UHRF1 assumes a ‘closed’ conformation with diminished E3 ligase activity when the TTD domain interacts with the internal PBR^32–35^. To identify compounds that block this internal TTD:PBR interaction, we developed an assay using AlphaScreen (Amplified Luminescent Proximity Homogeneous Assay, Perkin Elmer) technology that detects the interaction between biotinylated-AviTag-N-UHRF1 (containing UBL and TTD, aa 1–285) and FLAG-C-UHRF1 (containing SRA, RING E3 domains and PBR, aa 414–793) (Fig. 1A,C). We confirmed that the N-UHRF1:C-UHRF1 interaction is TTD-dependent by showing that H3K9me3 peptide, but not H3K4me3 peptide, could inhibit the interaction (Supplementary Fig. S1). The signal change in each well was calculated relative to DMSO controls on each plate. Using a 3 standard deviation (> 31.7% inhibition) cut off, we identified 145 fragments (6.2% hit rate) that inhibited the N-UHRF1:C-UHRF1 interaction (Fig. 1B).

Finally, the fragment library was screened using a TR-FRET assay to find inhibitors that block the interaction of His_6_-TTD with H3K9me3 peptide. In this assay, an anti-His_6_-antibody labeled with Tb cryptate binds to His_6_-TTD while a streptavidin-d2 acceptor binds to a biotinylated histone H3K9me3 peptide. The binding between His_6_-TTD and H3K9me3 peptide was measured by the ratio of 665 nm fluorescence (from the d2 acceptor) to 620 nm fluorescence (from the Tb cryptate donor) (Fig. 1D). The signal change in each well was calculated relative to the DMSO control on each plate with 3 standard deviations (> 24% inhibition) being used as the cut off. This led to the identification of 101 fragments (4.3% hit rate) that inhibited the interaction of the His_6_-TTD with H3K9me3 peptide (Fig. 1B).

It has previously been reported that when employing more than one fragment screening assay, a significant portion of hits identified in each screen may not overlap with the others^27,50–52^, and we have similar observations in our study. In one previous study, a 361-fragment library was screened against an aspartic protease, endothiapepsin, by six different screening methods: saturation-transfer difference NMR, a fluorescence-based biochemical screen, a reporter-displacement assay, native mass spectrometry, microscale thermophoresis, and TSA^50,51^. Out of the 361 fragments, two-thirds of the entire library were identified by at least one assay, 41 hits were detected by at least two assays, but no hit was identified by all assays. This underscores the utility of using binding-based and/or functional screens to triage hits. If performed in isolation, each of the screens used depicts a different scenario given that the majority of the hits within each of the screens were nonoverlapping with another assay. For example, in our work, of the 175 hits that were detected by TSA, only 9.1% confirmed activity in at least one function-based screen (AlphaScreen or TR-FRET), suggesting that the TTD has alternative binding sites located distally to the site that mediates histone or PBR binding, or that TSA is prone to false positive results. Given our goal to develop chemical probes to perturb these PPIs, TSA alone would not have been an insightful assay since it was only 9.1% successful in identifying functionally active binders. Additionally, of the fragments that scored as hits in the AlphaScreen or TR-FRET function-based assays, only 15 hits were common to both, indicating that having a single activity does not guarantee the other. Again, either of the function-based screens alone would only have been ~ 10–15% informative of fragments having both functions. Somewhat surprising was the fact that only a small subset of the AlphaScreen and TR-FRET fragment hits (8.9% and 4.9% respectively) scored as hits in the TSA. Since TSA measures binding in a functionally independent context, this non-concordance might imply that: 1) the AlphaScreen and TR-FRET screens both have high false positive rates, 2) the TSA for TTD is prone to false positive interactions that increase the melting temperature, or 3) certain binding modes detected by the AlphaScreen or TR-FRET assays do not result in stabilization in the TSA, resulting in false negatives. Determining which of these scenarios (or combinations) is operative for deciding follow-up would be confounding; however, the situation is mitigated by considering the overlap of the hits in all 3 assays. We chose the highest level of stringency for follow-up: fragments that stabilized UHRF1-TTD (TSA), inhibited the N-UHRF1 from interacting with the PBR in C-UHRF1 (AlphaScreen) and disrupted the TTD interaction with histones (TR-FRET). We recommend the use of binding-based assays and function-based assays in parallel to prosecute fragment screening for targets involving PPIs.

**Figure 2:**
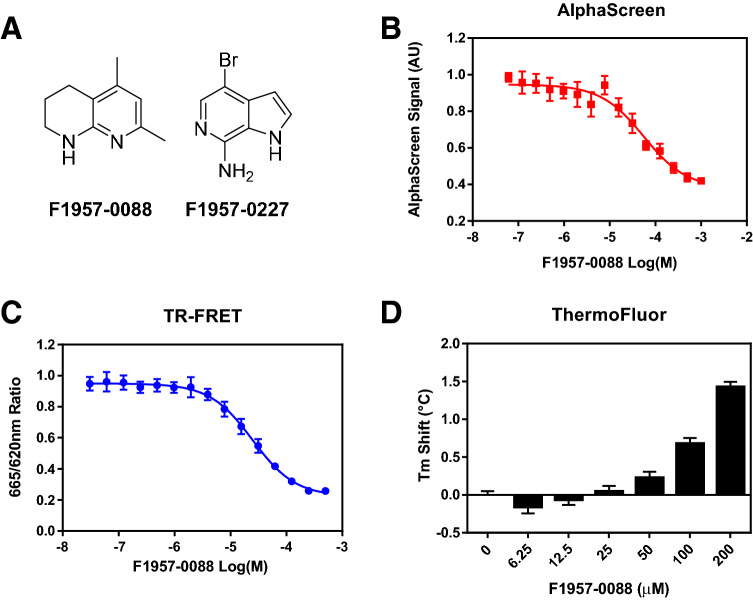
A fragment hit (F1957-0088) dose-dependently targets TTD in three parallel assays. (**A**) Chemical structures of two fragment hits that overlapped in all three screening methods. (**B**) F1957-0088 dose-dependently inhibited N-UHRF1 interaction with C-UHRF1 using AlphaScreen assay. (**C**) F1957-0088 dose-dependently inhibited His-TTD binding to H3K9me3 peptide in TR-FRET assay. (**D**) F1957-0088 dose-dependently increased the melting temperature (Tm) of His-TTD using the TSA. n = 3 for AlphaScreen, TR-FRET, and TSA and the error bars represent standard deviation.

Two fragments (compounds F1957-0088 and F1957-0227) scored as hits in all three assays (Fig. 2A and Supplemental Table S1). F1957-0227 was identified as a hit in two other AlphaScreen assays (N-UHRF1 vs. USP7-UBL1/2 and UHRF1-SRA domain vs. DNMT1-RFTS domain) and in three TSAs against unrelated protein targets (USP7-UBL1/2, UHRF1-SRA domain, and DNMT1-RFTS domain); we concluded that F1957-0227 behaves as a pan-assay interference compound (PAINS)^53^ and is thus not suitable for follow-up. Interestingly, although we used in-silico filters and manual structure evaluation to remove known examples of PAINS when constructing our fragment library, we still observed compounds that were active across different assays. As our example shows, in any screening campaign care must be taken to track the performance of individual hits throughout the lifetime of the library, as it is unreasonable to expect an in-silico filter to identify a priori all PAINS for all screening methods.

We analyzed the screening data for structure activity relationships (SAR) and found a fragment homolog, F1957-0202, that has the same bicyclic 1,2,3,4-tetrahydro-1,8-naphthyridine scaffold as F1957-0088, the only difference being 5,7-dimethyl groups on F1957-0088 compared to a 6-bromo group on F1957-0202 (Supplemental Table S1). These differences render F1957-0202 unable to inhibit His_6_-TTD binding to H3K9me3 peptide or stabilize His_6_-TTD in TSA, but it can inhibit the interaction between N-UHRF1 and C-UHRF1 (Supplemental Table S1), suggesting that there might be differences between the compound binding pocket on isolated His_6_-TTD and the TTD within N-UHRF1. We postulate that different modifications on the bicyclic 1,2,3,4-tetrahydro-1,8-naphthyridine scaffold may determine whether the fragment is active against His_6_-TTD:H3K9me3 and/or N-UHRF1:C-UHRF1 interaction. In the end, we selected compound F1957-0088 for further characterization. We repurchased the fragment and verified its dose-dependent activities using the AlphaScreen, TR-FRET, and TSA assays. F1957-0088 showed dose-dependent inhibition in both AlphaScreen (EC_50_ = 56.7 ± 6.6 µM) and TR-FRET (EC_50_ = 45.1 ± 5.46 µM) assays (Fig. 2B,C, and Table 1). F1957-0088 also stabilizes His_6_-TTD in a dose-dependent manner (Fig. 2D).

**Table 1:** The structure activity relationship (SAR) of F1957-0088 homologs with fused-bicyclic ring system.

Compound ID	Compound structure	TR-FRET (TTD vs. H3K9me3) EC_50_ (μM)	AlphaScreen (N-UHRF1 vs. C-UHRF1) EC_50_ (μM)	TSA (ΔT_m_) at 500 μM
F1957-0088		45.1 ± 5.4	56.7 ± 6.6	1.52 ± 0.09
**1**		> 500	56.7 ± 6.6	56.7 ± 6.6
**2**		109 ± 18.2	164.0 ± 33.0	0.75 ± 0.02
**3**		58.5 ± 8.4	75.7 ± 14.4	0.1 ± 0.06
**4**		> 500	> 500	− 0.23 ± 0.06
**5**		No inhibition	275.2 ± 174.8	0.51 ± 0.06
**6**		> 500	281.7 ± 227.7	0.21 ± 0.06
**7**		> 500	379.8 ± 176.0	0.19 ± 0.06

The EC_50_ of TR-FRET and AlphaScreen assays are reported as EC_50_ ± S.E.M. and the ΔT_m_ of TSA assay are reported as mean ± S.D. n=3 for AlphaScreen, TR-FRET, and TSA. Note that “no inhibition” means little to no change in assay readout even at the highest compound concentration tested (1 mM) while “> 500” means there is assay signal reduction/inhibition at highest concentrations yet the EC_50_ determined by curve fitting is above 500 µM.

### SAR of F1957-0088 homologs identify 2,4-lutidine as a potent fragment against UHRF1-TTD

Probing the structure–activity relationships (SAR) for a fragment hit is needed to validate the hit, identify the chemical moieties that drive binding, and set the stage for future optimization efforts. We purchased 16 structural variants of F1957-0088 and analyzed their effects on the N-UHRF1:C-UHRF1 interaction, TTD:H3K9me3 peptide interaction, and TTD melting temperature (Tables 1 and 2). Of these 16 compounds, 7 had similar fused bicyclic ring systems as F1957-0088 (Table 1) while 9 were substituted pyridine compounds (Table 2). All the fused bicyclic homologs were less potent than F1957-0088 in all three assays (Table 1). Compound **1**, with pyrazol fused to the 2,4-dimethylpyridine core, interacts much weaker in all three assays, suggesting that the F1957-0088 binding pose is incompatible with this replacement of the piperidine ring. Removal of both pyridine methyl groups (**2**) decreases inhibition in both interaction assays, but removal of the 2-methyl and addition of a 3-bromo group has a more modest effect (comparing **2** and **3**). Moving the 4-methyl group from the pyridine to the piperidine ring abolishes interaction in all assays (comparing **3** and **4**). Together, compounds **2**–**5** show that the location of substituents on the 1,2,3,4-tetrahydro-1,8-naphthyridine modulate the inhibition of TTD:H3K9me3 and N-UHRF1:C-UHRF1 interactions, suggesting shape complementarity between TTD and the best binders and implicating a piperidine methyl-mediated clash in the failure of **4** to bind (**4** is racemic, so apparently neither enantiomer is capable of binding). The importance of the amino group in the fused piperidine ring is confirmed by the greatly decreased potency of 5,6,7,8-tetra-hydro-quinoline **6**. However, adding another amino group to give 1,2,3,4-tetrahydropyrido[2,3-b]pyrazine **7** also substantially diminishes inhibitory activity.

**Table 2:** The SAR of F1957-0088 homologs with single pyridine ring

Compound ID	Compound structure	TR-FRET (His_6_-TTD vs. H3K9me3) EC_50_ (μM)	AlphaScreen (N-UHRF1 vs. C-UHRF1) EC_50_ (μM)	TSA (ΔT_m_) at 500 μM
2,4-lutidine		29.2 ± 1.4	10 ± 2.1	2.04 ± 0.01
**8**		238.4 ± 89.1	52.6 ± 6.9	0.66 ± 0.09
**9**		No inhibition	No inhibition	− 0.01 ± 0.06
**10**		81.9 ± 8.5	20.2 ± 2.1	1.14 ± 0.06
**11**		322.8 ± 79.2	No inhibition	0.17 ± 0.06
**12**		51.9 ± 8.1	21.3 ± 3.5	1.59 ± 0.06
**13**		258.7 ± 117.5	> 500	0.33 ± 0.06
**14**		42.5 ± 4.4	> 500	0.89 ± 0.06
**15**		47.2 ± 4.9	> 500	1.11 ± 0.06

The EC_50_ of TR-FRET and AlphaScreen assays are reported as EC_50_ ± S.E.M. and the ΔT_m_ of TSA assay is reported as mean ± S.D. n=3 for AlphaScreen, TR-FRET, and TSA. Note that “no inhibition” means little to no change in assay readout even at the highest compound concentration tested (1mM) while “>500” means there is assay signal reduction/inhibition at highest concentrations yet the EC_50_ determined by curve fitting is above 500 µM.

Our SAR analysis of hit F1957-0088 includes the single ring pyridine homologs, and 2,4-lutidine emerged as the most potent inhibitor identified in both TR-FRET (EC_50_ = 29.2 ± 1.4 µM) and AlphaScreen (EC_50_ = 10.0 ± 2.1 µM) assays (Table 2). The 2-methyl group on the pyridine ring is important, since changing it to a 2-amino group (**8**) raises the EC_50_ as assessed by both TR-FRET (238.4 ± 89.1 µM) and AlphaScreen (52.6 ± 6.9 µM), while changing to a 2-chloro group (fragment **9**) completely abolishes activity. Comparing fragments **10** and **11** reveals that having a methyl group adjacent to the nitrogen on the pyridine ring (compound **10**) ensures higher inhibitory activity than a 4-methyl group (compound **11**) in the context of the same exocyclic amino group. Compared to 2,4-lutidine, fragment **12** has a slightly higher EC_50_ in TR-FRET (51.9 ± 8.1 µM) and AlphaScreen (21.3 ± 3.5 µM) assays, suggesting that placing an amino group adjacent to the nitrogen in 2,4-lutidine is well tolerated and can be a potential location for further modification of 2,4-lutidine. When the TR-FRET activity of compounds **13** and **14** are compared, it is seen that adding a 5-chloro group is detrimental to the inhibitory effect against the TTD-H3K9me3 interaction whereas a 5-methyl group is tolerated. Similarly, having a methyl group at the 2, 4, and 6 positions does not strongly impact its TR-FRET EC_50_. Although the trend of changes in TR-FRET and AlphaScreen EC_50_ were usually congruous, fragments **14** and **15** inhibited TTD-H3K9me3 interaction with reasonably strong EC_50_ values of 42.5 ± 4.4 µM and 47.2 ± 4.9 µM respectively in the TR-FRET assay but lost almost all their inhibitory activity in the AlphaScreen assay. Since the TR-FRET assay probes the TTD:H3K9me3 peptide interaction and the AlphaScreen assay detects the binding between N-UHRF1 and C-UHRF1, it may be possible to selectively block UHRF1 histone binding without altering its conformational state. Based on the F1957-0088 analogs examined, we selected 2,4-lutidine due to its higher potency for further characterization by both protein NMR and X-ray crystallography to probe the details of the interaction.

### NMR chemical shift perturbations show that 2,4-lutidine affects sites throughout the TTD

**Figure 3:**
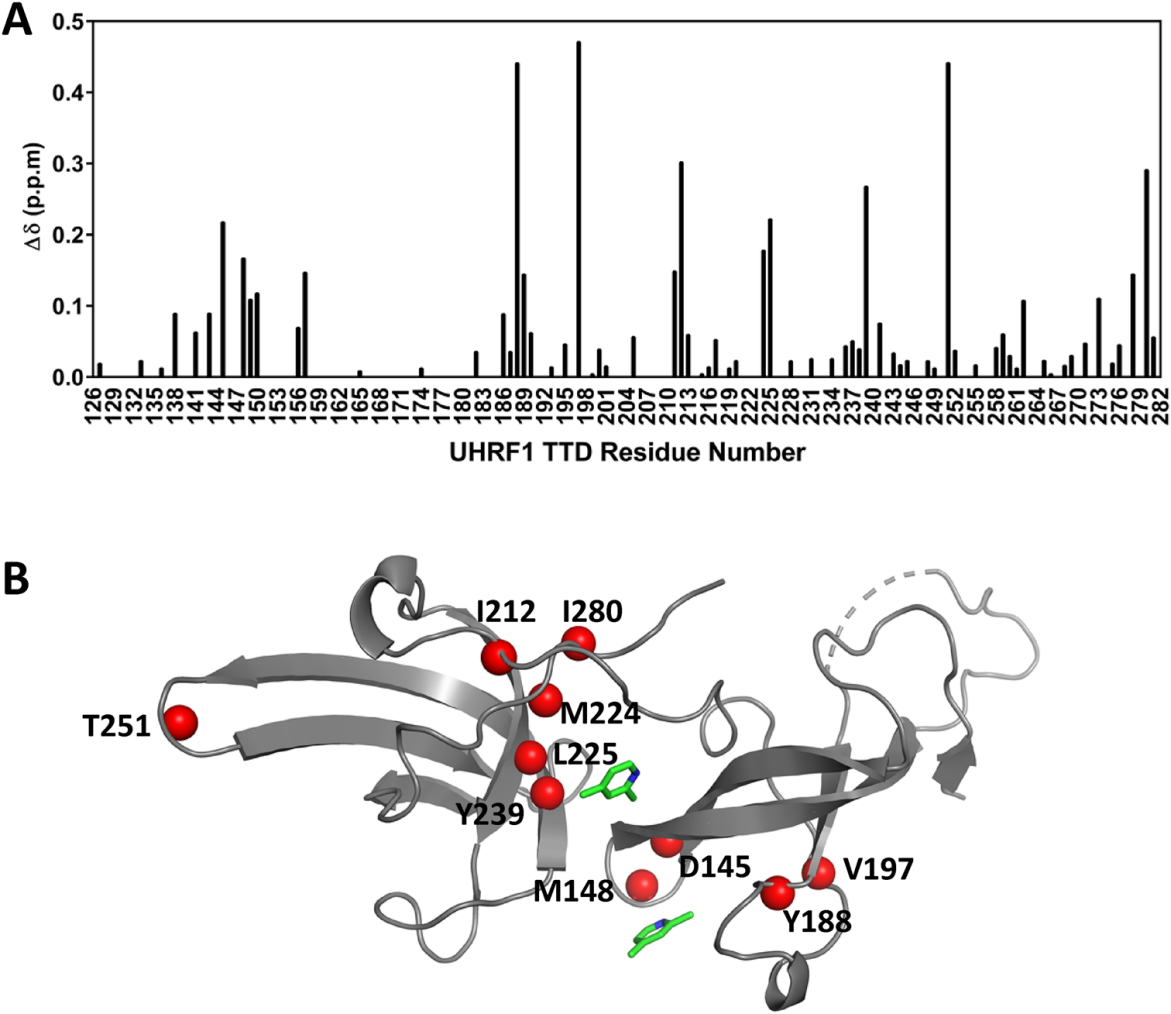
2,4-lutidine induces NMR chemical shift changes in UHRF1-TTD. (**A**) 2,4-lutidine induces chemical shift changes throughout the TTD sequence. We detect five additional strongly perturbed resonances (Δδ > 0.2) that were not assigned previously and thus not included in this graph. (**B**) Mapping the 10 largest chemical shift changes onto the TTD structure (red spheres) shows that most are located close to the interface between the two Tudor domains. The structure shown here is the co-crystal structure of TTD/2,4-lutidine (PDB ID: 6VYJ).

We obtained structural evidence for the binding of 2,4-lutidine to TTD in solution using NMR chemical shift perturbations. ^1^H-^15^N HSQC spectra of 300 µM ^15^N-labeled TTD acquired in buffer or with 2,4-lutidine concentrations ranging from 55 to 588 µM revealed considerable systematic chemical shift changes (Δδ) for about one third of the resolved resonances (Fig. 3A and Supplementary Fig. S2). Using previously published resonance assignments^9^, we identified 72 backbone amides and one tryptophan indole NH for which chemical shifts could be reliably assigned in the apo state and chemical shift changes could be measured (Fig. 3A). Chemical shift changes occur at sites distributed along the sequence and across the structure of the TTD, indicating that the binding of 2,4-lutidine influences average backbone conformation throughout the TTD. Because nine of the ten most perturbed (assigned) peaks are located near the interface between the Tudor domains (Fig. 3B), we infer that binding of 2,4-lutidine modulates the interaction between the two Tudor domains. This inference is consistent with the ability of 2,4-lutidine to increase the melting temperature of TTD by 2.04 ± 0.01 °C (Table 2). Chemical shift changes of the five most strongly affected resonances fit to a single site binding model with a K_d_ of 31 µM (Supplementary Fig. S3). The most strongly perturbed resonance dominates this fit; excluding it gives a best-fit K_d_ of 38 µM. These values indicate high ligand efficiency: K_d_ values of 31 or 38 µM correspond to ligand efficiencies of 0.79 or 0.77. The ligand efficiency is calculated by dividing the binding free energy of a ligand by its number of heavy (non-hydrogen) atoms and it is commonly used in FBLD as a metric to compare the degree to which a fragment is optimally binding to its target^54^. A fragment that possesses high ligand efficiency can serve as a good starting point for optimization to obtain high affinity ligands^55^.

### The co-crystal structure of 2,4-lutidine binding to TTD

**Figure 4:**
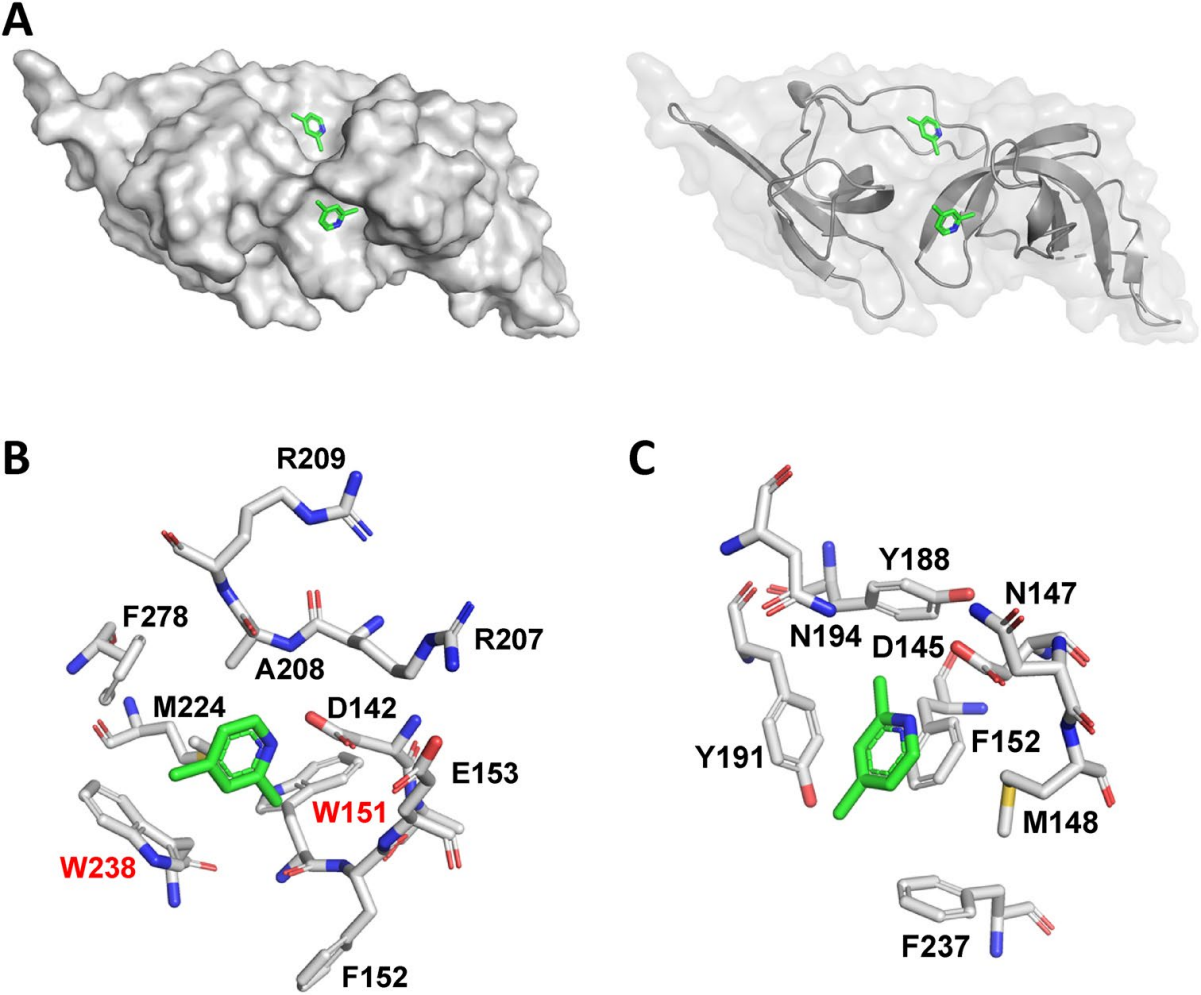
X-ray co-crystal structure elucidates the binding mode of 2,4-lutidine to TTD. (**A**) 2,4-Lutidine binds to two sites on TTD. (**B**) 2,4-Lutidine binds to the Arg-binding cavity of TTD. The two tryptophans near the 2,4-lutidine binding site, W151 and W238 are labeled in red (**C**) 2,4-Lutidine also binds to the aromatic cage of TTD.

Our co-crystal structure of TTD and 2,4-lutidine at 1.4 Å resolution reveals that 2,4-lutidine binds to two sites on UHRF1-TTD (Fig. 4A, Supplementary Fig S4A, and Supplementary Table S2): the aromatic cage that interacts with trimethylated lysine^9,10,39^ and the Arg-binding cavity^10,12,33,39^. Both sites are known to play crucial roles in binding to H3K9me3 peptide, the PBR, the L2 linker, and LIG1^9–12,33,39^. When 2,4-lutidine binds to the Arg-binding cavity, it favorably interacts with Trp238 and Phe278 through π–π T-shaped interaction (Supplementary Fig. S5A) and makes π-alkyl hydrophobic interactions with Ala208 and Met224 (Fig. 4B and Supplementary Fig. S5B). The distance between the nitrogen on 2,4-lutidine and the carboxylate group of Asp142 is 2.8–3.1 Å (Supplementary Fig. S4B) and these atoms likely form a hydrogen bond or salt bridge that stabilizes the fragment:TTD interaction (Fig. 4B and Supplementary Fig. S4B), since the 2,4-lutidine pKa is 6.99 (note that in other complexes, Asp142 interacts via hydrogen bonds with Arg649 of PBR^33^, Arg296 of TTD-PHD linker^10,12^, or Arg121 of LIG1^39^). The second 2,4-lutidine molecule in the structure occupies the aromatic cage that is known to bind the trimethyl lysine on histone H3 (H3K9me3) and LIG1 (K126me3)^9,10,12,39^ or Pro656 of the PBR^33^. In our structure, 2,4-lutidine interacts with Phe152 through π–π stacking (Supplementary Fig. S5C) while contacting Tyr191 and Phe237 via π–π T-shaped interaction and making hydrophobic interactions with Tyr188 (Fig. 4C and and Supplementary Fig. S5D). Additionally, it may form a weak hydrogen bond or salt bridge with Asp145, as the distance between the 2,4-lutidine nitrogen and the carboxylate group of Asp145 is 3.2–3.8 Å (Supplementary Fig. S4C). In summary, based on the crystal structure, TTD binds 2,4-lutidine with two binding pockets that are each important for its interaction with histone H3, Linker L2, PBR, and LIG1. This structure, involving two-site binding, may explain why 2,4-lutidine was active not only in the thermal shift assay, but also inhibited the TTD interaction with both PBR and histone peptides in the AlphaScreen and TR-FRET assays. It may further explain why most of the fragment hits identified possessed only one of two functional activities, indicating that they may bind to one of the pockets preferentially.

### Using intrinsic tryptophan fluorescence to monitor 2,4-lutidine binding to TTD

**Figure 5:**
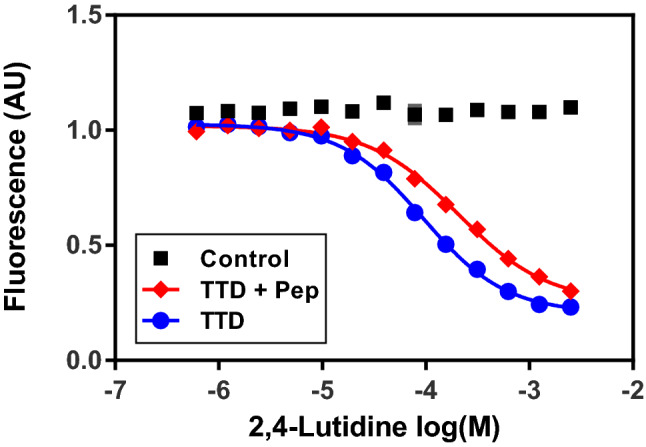
Tryptophan fluorescence monitors 2,4-lutidine binding to TTD. 2,4-Lutidine dose-dependently quenched the tryptophan fluorescence of TTD (blue circle), and adding H3K9me3 (1–20) peptide to twofold final TTD concentration (red diamond) shift the apparent K_d_ of 2,4-lutidine binding from 98.2 ± 3.0 to 237.3 ± 11.4 µM (shown as K_d_ ± S.E.M). 2,4-Lutidine did not inhibit the tryptophan fluorescence of control protein (black square, His-USP7-UBL1/2). n = 3 for each data point. *Note*: the standard deviation error bars are included, but the error values are often extremely small in this assay.

Our co-crystal structure revealed two tryptophan residues (Trp151 and Trp238) located close to the Arg-binding cavity where 2,4-lutidine binds (Fig. 4B). Although the NMR chemical shifts for the Trp238 amide are not known, the amide resonances of Trp151 and of Tyr239 are resolved and significantly perturbed by 2,4-lutidine titration (Supplementary Fig. S2). The fluorescence of tryptophan is sensitive to the polarity of its local environment and can thus be used to monitor ligand binding or conformational changes^56,57^. We hypothesized that 2,4-lutidine may affect TTD intrinsic tryptophan fluorescence upon binding to the Arg-binding cavity. After optimizing the intrinsic fluorescence assay (Supplementary Fig. S6), the dose-dependent effect of 2,4-lutidine on TTD intrinsic fluorescence emission was monitored at 334 nm (excitation at 280 nm) with 15 µM TTD protein. 2,4-lutidine quenches TTD fluorescence in a dose-dependent manner, with an apparent K_d_ of 98.2 ± 3.0 µM (Fig. 5, K_d_ ± S.E.M.). The higher K_d_ value measured here (compared to that from NMR chemical shift perturbations) suggests that the fluorescence changes may report on 2,4-lutidine binding to both the Arg-binding cavity and the aromatic cage; if this is the case, the K_d_ from fluorescence represents an average over the two known binding sites. This higher K_d_ value nevertheless corresponds to a high ligand efficiency of 0.70. Adding 30 µM H3K9me3 peptide (a two-fold molar excess over TTD) shifts the K_d_ of 2,4-lutidine to 237.3 ± 11.4 µM (Fig. 5, K_d_ ± S.E.M.), suggesting that 2,4-lutidine competes with H3K9me3 peptide for binding to TTD.

#### 2,4-Lutidine inhibits H3K9me3 peptide binding to TTD-PHD and full length UHRF1

**Figure 6:**
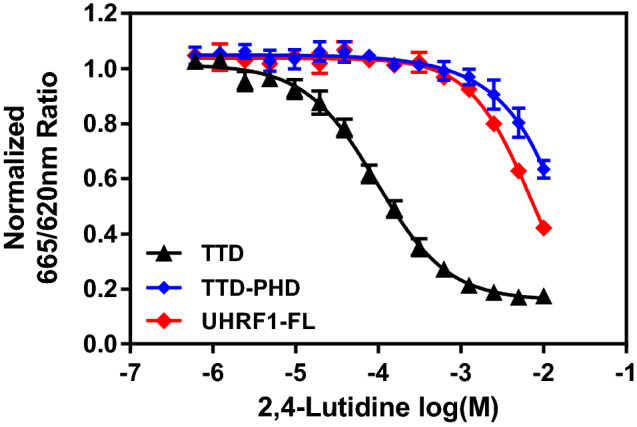
2,4-Lutidine also inhibits TTD-PHD and UHRF1-FL activities. The TR-FRET assay indicated 2,4-lutidine only started to inhibit the binding of TTD-PHD and UHRF1-FL to the H3K9me3 peptide at mM concentration.

Because all five UHRF1 domains are implicated in extensive interactions with one another^15^, we wished to determine the effect of 2,4-lutidine on interactions made by multi-domain and full-length UHRF1 constructs. We therefore measured the dose-dependent inhibition by 2,4-lutidine of His_6_-TTD-PHD (aa 123–366) and His_6_-UHRF1-full length (His_6_-UHRF1-FL) binding to biotinylated H3K9me3 peptide. In a side by side comparison, 2,4-lutidine strongly inhibits the His_6_-TTD interaction with H3K9me3 peptide but only weakly inhibits the His_6_-TTD-PHD or His_6_-UHRF1-FL interactions with the same H3K9me3 peptide (the mid-points are shifted about two logs; Fig. 6). 2,4-Lutidine inhibits His_6_-UHRF1-FL binding to the peptide slightly better than His_6_-TTD-PHD (Fig. 6). Given that the intrinsic tryptophan fluorescence assay works well to determine the K_d_ of 2,4-lutidine binding to TTD, we used this assay to characterize 2,4-lutidine binding to His_6_-TTD-PHD and UHRF1-FL with C-terminal FLAG tag (C-FLAG-UHRF1-FL; Supplemental Fig. S7). Similar to our TR-FRET assay results, only at close to mM concentrations did 2,4-lutidine start to quench the fluorescence of His_6_-TTD-PHD. In contrast, 2,4-lutidine did not quench the fluorescence of C-FLAG-UHRF1 at all. This reduction of activity is likely due to the intra-molecular interactions of these larger protein domains/regions, such as TTD:linker 2 and TTD:PBR interactions^10,12,33^, which may partially block 2,4-lutidine from interacting with the binding pocket(s) on TTD. This highlights the advantages of using truncated protein constructs such as TTD in FBLD, because using UHRF-FL in the fragment screen would not have identified 2,4-lutidine or its homologs as fragment hits. The fact that the 2,4-lutidine binding pockets on TTD are less accessible in UHRF1-FL suggest the binding surfaces are likely important for intraprotein interaction and may play crucial roles in UHRF1 biological functions. Being a small chemical fragment, 2,4-lutidine cannot block the intraprotein interactions effectively, but future optimized compounds based on the 2,4-lutidine scaffold may have the potential of regulating both the intramolecular and intermolecular interactions of UHRF1.

#### Protein kinase A treatment increased TTD-PHD sensitivity to 2,4-lutidine inhibition

**Figure 7:**
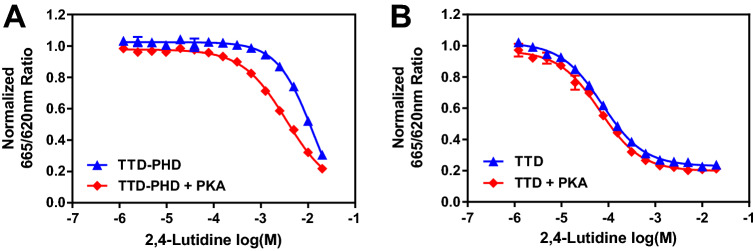
The PKA treatment of TTD-PHD improved 2,4-lutidine inhibition effect. (**A**) Treating TTD-PHD with PKA at 30 °C for 1 h improved 2,4-lutidine inhibition effect on its binding to H3K9me3 peptide in TR-FRET assay. (**B**) Same PKA treatment had no effect on 2,4-lutidine dose-dependent inhibition on TTD-H3K9me3 peptide interaction.

As stated previously, our crystal structure show that 2,4-lutidine binds to two sites on TTD: an aromatic cage that can bind the methylated lysine on the H3K9me3 peptide, and the Arg-binding cavity known to interact with the arginine of the linker between TTD and PHD domain^10,12^. We hypothesize that the higher concentration of 2,4-lutidine needed to inhibit TTD-PHD:H3K9me3 peptide interaction results from the blockage of one of the binding pockets (Arg binding cavity) by Arg296 of the TTD-PHD linker^10,12^. It has been reported that the phosphorylation of Ser298 on the linker region by protein kinase A can considerably decrease the TTD-linker interaction^10,58,59^. We hypothesize that when Ser298 is phosphorylated, the Arg-binding cavity will be more accessible for 2,4-lutidine binding. To explore this, we incubated His_6_-TTD and His_6_-TTD-PHD with or without protein kinase A (PKA) and ATP for 1 h at 30 °C to phosphorylate the proteins. After incubation, the dose-dependent inhibition by 2,4-lutidine of H3K9me3 peptide binding to the two proteins was tested using the TR-FRET assay. PKA treatment improves the ability of 2,4-lutidine to block His_6_-TTD-PHD binding to H3K9me3 peptide (Fig. 7A) but has no effect on its inhibition of His_6_-TTD binding to the peptide (Fig. 7B). To verify that His_6_-TTD-PHD is indeed phosphorylated by PKA and to identify the phosphorylation site(s), both PKA-treated and non-treated His_6_-TTD-PHD were digested with trypsin and the peptides were analyzed by liquid chromatography with tandem mass spectrometry (LC–MS/MS). After PKA treatment, the His_6_-TTD-PHD was 100% monophosphorylated, and the phosphorylation serine was mapped to the two serines on the peptide ^298^SGPSCK^303^, Ser298 or Ser301 (Supplemental Fig. S8), similar to previous reports^10,58,59^. The PKA mono-phosphorylation of His_6_-TTD-PHD at Ser298 (or Ser301) and the associated increase in sensitivity to 2,4-lutidine inhibition support our hypothesis that the L2 linker (aa. 285–317) between TTD and PHD may compete with 2,4-lutidine for the Arg binding cavity and render His_6_-TTD-PHD less sensitive to 2,4-lutidine inhibition.

#### TTD-PHD linker mutants are more sensitive to 2,4-lutidine inhibition

**Figure 8:**
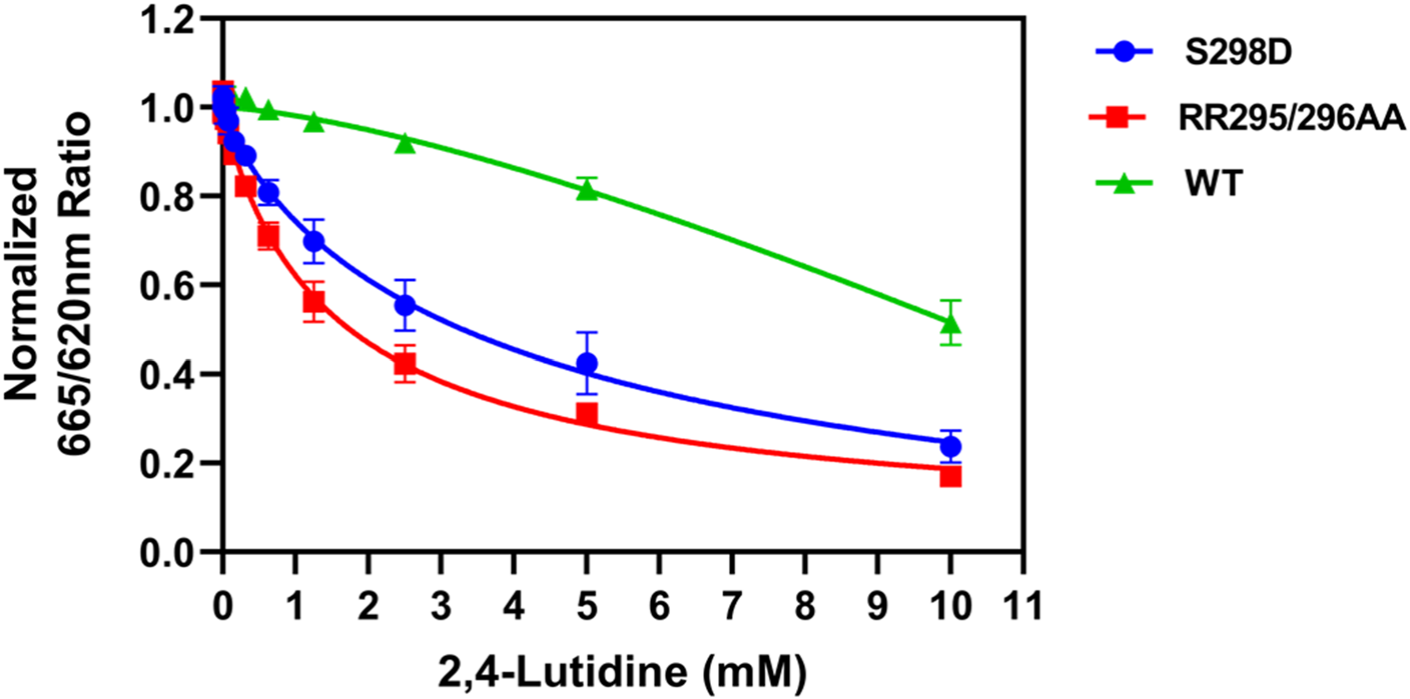
TTD-PHD linker mutants (S298D and RR295/296AA) are more sensitive to 2,4-lutidine inhibition. The TTD-PHD S298D mutant mimics Ser298 phosphorylation while the RR295/296AA mutates at the crucial sites that bind to the Arg binding groove of TTD. Both mutants damaged the interaction between the linker and the Arg-binding pocket. Using TR-FRET assay to characterize the 2,4-lutidine dose-dependent inhibition effect on the TTD-PHD:H3K9me3 peptide interaction clearly showed that the S298D and RR295/296AA mutants were more sensitive to 2,4-lutidine comparing to wild type (WT).

Since the PKA-treated, linker phosphorylated His_6_-TTD-PHD became more sensitive to 2,4-lutidine inhibition of its interaction with H3K9me3 peptide, we hypothesized that 2,4-lutidine might better inhibit His_6_-TTD-PHD carrying mutations that have been reported to damage the linker:Arg-binding cavity interaction^10,12,58,59^. To test this hypothesis, we generated two TTD-PHD mutants: (1) S298D, which mimics phosphorylation at Ser298 and (2) RR295/296AA, which prevents Arg295 and Arg296 of the TTD-PHD linker from contacting the Arg binding cavity of TTD. Dose-dependent 2,4-lutidine inhibition of wild type, S298D, and RR295/296AA interaction with H3K9me3 peptide using the TR-FRET assay shows that the two TTD-PHD mutants were each significantly more sensitive to 2,4-lutidine inhibition than wild type (Fig. 8). At 2.5 mM 2,4-lutidine, the binding of S298D and RR295/296AA mutant to H3K9me3 peptide were 44.5% and 57.6% inhibited, while wild type was only 7.9% inhibited. The EC_50_ of 2,4-lutidine inhibition of wild type TTD-PHD:H3K9me3 interaction was not determined due to non-saturating inhibition, whereas the EC_50_ of 2,4-lutidine against S298D or RR295/296AA were determined to be 4.3 ± 1.3 or 1.6 ± 0.2 mM (EC_50_ ± s.e.m.), respectively. In summary, these results support our hypothesis that the TTD-PHD linker competes with 2,4-lutidine for access to the Arg-binding pocket and thereby renders TTD-PHD less sensitive than TTD to the compound.

#### Discussion of potential lead optimization strategy: fragment linking

The distance between the 2,4-lutidine binding sites on the TTD is about 12 Å (Fig. S4A), suggesting that fragment-linking may be a viable strategy for generating more potent compounds. Fragment linking was first reported by Fesik and co-workers, who linked two weakly bound ligands (K_d_ values of 17 mM and 20 µM) to create a 15 nM inhibitor to a matrix metalloproteinase stromelysin^60,61^. The biggest challenge for this strategy is to maintain the correct binding mode of both linked fragments in the final compound^47,62^. Our studies suggest that 2,4-lutidine inhibits TTD activity at lower concentrations mostly via its binding to the Arg-binding cavity. In the TTD-PHD construct, when the linker usually binds to the Arg-binding cavity^10,12^, 2,4-lutidine can only inhibit at mM concentration (Fig. 6). Since the TTD co-crystal was obtained at 20 mM 2,4-lutidine, it is likely that 2,4-lutidine binds to the Arg-binding cavity with higher affinity than to the aromatic cage. Our NMR data also suggest 2,4-lutidine binds to one site with higher affinity (Supplementary Fig. S3). To generate a more potent linked inhibitor of the TTD, a fragment screen against a longer TTD construct that includes the L2 linker (TTD-linker), which serves as an intrinsic plug to the Arg-binding cavity, might reveal fragments that bind with higher affinity to the aromatic cage. The ligands for the aromatic cage and Arg-binding cavity could then be connected by spacers with different lengths to determine one that allows for optimal positioning of both 2,4-lutidine moieties.

## Conclusion

UHRF1 plays important yet complicated roles in cancer development and numerous studies have indicated that its silencing boosts tumor suppressor gene expression, causes cell cycle arrest, and triggers cancer cell apoptosis^20–24,63–67^. Despite being an attractive drug target, no highly potent UHRF1-targeting cancer therapeutics or chemical probes have been developed to date. This is likely because most UHRF1 domains function via protein–protein or protein-DNA interactions and are not traditional enzymatic drug targets. Moreover, it is not clear which of its five domains should be inhibited for cancer treatment.

In this study, we chose to target TTD over the other domains because we believed this domain might offer an opportunity for more selective inhibition while maintaining the critical housekeeping function—recognition of hemimethylated DNA by SRA domain during DNA replication. We used both binding and functional screens in parallel to identify fragments that inhibit the PBR and H3K9me3 peptide binding activities of UHRF1 TTD. Using the criteria that the fragments demonstrate activity in all three of our assays, we identified the molecule 2,4-lutidine as a moderately potent inhibitor of these activities with high ligand efficiency. Solving the co-crystal structure, we identified two binding pockets for 2,4-lutidine—the Arg-binding cavity and the aromatic cage. 2,4-Lutidine inhibits TTD binding to H3K9me3 peptide at µM levels, but shows only hints of the same activity on full length UHRF1 at mM concentrations. Clearly, further medicinal chemistry-based optimization is required for obtaining a biologically active TTD-targeting inhibitor of UHRF1. Given the proximity of the two binding sites, future success for targeting UHRF1-TTD may rely on linking optimized ligands bound to each of these two sites. Since the TTD is responsible for recognizing the H3K9me3 inhibitory histone marks, we envision potent TTD inhibitors may stimulate tumor suppressor genes expression through blocking UHRF1 from binding to the H3K9me3 inhibitory marks on their promoters. The FBLD approaches described here provides a path to such chemical probes and our progress toward their development will be reported in due course.

## Methods

### Life chemical fragment library construction

Our fragment library was purchased from Life Chemicals in an iterative fragment selection process, which started with the Life Chemicals team applying several filters to their fragment inventory. We requested a set of expanded rule-of-3 filters (Supplementary Table S3), followed by the removal of known pan-assay interference compounds (PAINS), compounds with multiple undefined stereocenters, electron-rich 5-membered heterocycles containing one heteroatom, electron rich anilines, Michael acceptors, long chain (> 3C) analogs, catechols, 4-F-phenylsulfonyl compounds, and N-Boc compounds, resulting in a list of 4800 fragments. At our request, this was subsequently reduced to a diversity set of 3000 fragments by Life Chemicals, and each structure was manually inspected and approved by our in-house medicinal chemists. Fragments that failed this inspection were replaced. We then selected the final screening library of 2500 fragments using BIOVIA Pipeline Pilot^68^: 42 molecular quantum numbers (MQNs)^69^ were calculated for each fragment, and these formed the basis for grouping the fragment library into 2500 clusters; the fragment at the center of each cluster was selected for purchase. Upon delivery, the fragments—in racks containing 96 Thermo Scientific Matrix Alphanumeric Storage Tubes—were dissolved in DMSO-d6 using a Tecan Freedom EVO 200 Liquid Handling System. The 2325 successfully solubilized fragments were transferred to 384-deep well plates, sealed under argon gas using Agilent PlateLoc thermal microplate sealer, and protected from UV light by storing in amber desiccators filled with nitrogen gas at room temperature.

### Protein expression and purification

The TTD, TTD-PHD, FLAG tagged-C-UHRF1, and UHRF1-FL were cloned into the pMCSG7 vector and these His-tagged proteins were expressed in Rosetta (DE3) *E. coli*. The N-UHRF1 was cloned into pMCSG51, and the His-AviTag-N-UHRF1 was expressed in Rosetta (DE3) *E. coli* cells (induced at 18 °C overnight with 50 µM biotin and 100 µM IPTG). The His-AviTag-N-UHRF1 was specifically biotinylated on its AviTag by the BirA that was on the same vector (pMCSG51). His_6_-TTD and His-AviTag-N-UHRF1 were purified by Nuvia IMAC (Ni) column (BioRad) and anion exchange Foresight Q column (BioRad) using an NGC medium-pressure chromatography system (BioRad). His_6_-TTD-PHD was purified by Nuvia IMAC (Ni) column (BioRad). Since the purity of the pooled fractions of His_6_-TTD-PHD (based on gel image) is ≥ 90%, we chose to omit the use of exchange Foresight Q column. Similarly, His_6_-TTD-PHD Ser298 and His_6_-TTD-PHD RR295/296AA were also purified by Nuvia IMAC (Ni) column (BioRad). The His-UHRF1-FL was purified by HisPur Ni–NTA Resin (ThermoFisher). The His_6_-C-FLAG-UHRF1 was first purified by Nuvia IMAC column followed with TEV protease digestion to remove the His-tag, and the FLAG-C-UHRF1 was further purified by collecting unbound fractions through another run of the Nuvia IMAC column. Because the TEV protease cleavage following with another pass through Nuvia IMAC column removed most of the impurities, we did not include the ion exchange purification for C-FLAG-UHRF1. The purified proteins were concentrated by ultrafiltration and aliquoted and stored in − 80 °C freezer until use. The protein concentration was determined using OD_280_ from a Denovix DS-11 spectrometer based on the extinction coefficient and molecular weight of each protein.

### Thermal shift assay (TSA) and fragment screen

The fragment screen was carried out on LightCycler 480 Multiwell Plates 384 (Roche). The TSA mix contains 0.15 mg/ml of His_6_-TTD, 10 × of SYPRO Orange dye (ThermoFisher) in the TSA buffer (50 mM sodium phosphate, pH = 7, 300 mM NaCl). During the screen, 5 µl of TSA mix was dispensed into each well, and 100 nl of chemical fragments stock (10 mM in DMSO) was added to each well by pintool using Tecan Freedom Evo liquid handler to make the final compound concentration 200 µM. After 1 h of incubation at room temperature, the increase of fluorescence at 580 nm (excitation 465 nm) during the 20–90 degree melting (0.04 °C/s) was measured by LightCycler 480 Instrument II (Roche). For the fragment screen, on seven of the library plates there were 32-wells of DMSO control, while there were 8-wells of DMSO control in the last plate. To characterize the SAR of F1957-0088 homologs (in Tables 1 and 2), same protein and dye concentration was used, only that the TTD without his-tag (TEV protease cleaved) and phosphate buffer with lower NaCl concentration was used (50 mM sodium phosphate, pH 7, 150 mM NaCl). Using un-tagged TTD for TSA provide closer resemblance to the native state of TTD. The lower salt concentration (150 mM NaCl) also allowed better comparison across three different assays during the dose-dependent SAR studies as both AlphaScreen and TR-FRET assay also use buffers containing 150 mM NaCl.

### AlphaScreen assay and fragment screen

The fragment screen was carried out on low volume 384-well white plates (Corning 4512) and the assay was performed at room temperature. First, 5 µl of AlphaScreen buffer (50 mM HEPES, pH = 6.5, 150 mM NaCl, 0.1% Triton X-100, 1 mM DTT) was dispensed by multidrop combi microplate dispenser (Thermo Scientific) and 300 nl of chemical fragment stock (10 mM in DMSO) was added to each well by pintool using Tecan Freedom Evo liquid handler. Next, 3 µl of AlphaScreen reagent mix containing 768 nM of Biotinylated-His-Avitag-N-UHRF1, 768 nM of FLAG-C-UHRF1, 40 µg/ml streptavidin donor beads, and 40 µg/ml Anti-FLAG conjugated acceptor beads (PerkinElmer) was added to each well by multidrop. The final concentration of FLAG-C-UHRF1 was 300 nM, Biotinylated-His-Avitag-N-UHRF1 was 300 nM, Donor/acceptor beads each was 15 µg/ml. In this assay, the biotinylated-AviTag-N-UHRF1 binds to streptavidin-coated donor beads while the FLAG-C-UHRF1 interacts with the anti-FLAG antibody-coated acceptor beads. The final compound concentration was 360 µM. After 1 h of incubation, the AlphaScreen signal of each plate was measured with a Tecan Infinite M1000 plate reader, which uses a high-power laser (680 nm, 750 mW) as the excitation light source for the donor beads and the AlphaScreen filter was used to detect the emission between 520 and 620 nm from the acceptor beads. For the dose-dependent assays for SAR, the final concentrations of N-UHRF1, C-UHRF1, donor, and acceptor beads were the same as fragment screen. The only differences were that the phosphate buffer was used (50 mM sodium phosphate, pH = 7, 150 mM NaCl, 1 mM DTT, and 0.1% Triton-X-100) and the final volume was 10 µL. For the fragment screen, on seven of the library plates there were 32-wells of DMSO control, while there were 8-wells of DMSO control in the last plate.

### TR-FRET assay and fragment screen

The fragment screen was carried out on low volume 384-well white plates (Corning 4512) and the assay was performed at room temperature. The TR-FRET reagent mix contained 200 nM of biotinylated-H3K9me3 peptide (Anaspec), 250 nM of His_6_-TTD, 25 nM of streptavidin-d2 (CisBio), and 1 × anti-6His-Tb cryptate Gold (Cisbio) in TR-FRET buffer (50 mM sodium phosphate, pH 7, 150 mM NaCl, 1 mM DTT, and 0.1% Triton-X-100). During the screen, 5 µl of TR-FRET reagent mix was dispensed into each well, and 100 nl of chemical fragments stock (10 mM in DMSO) was added to each well by pintool using Tecan Freedom Evo liquid handler to make the final compound concentration 196 µM. After 1 h of incubation, the fluorescent signals at 665 nm and 620 nm of each plate were measured by the Tecan Infinite M1000 plate reader (excitation 340 nm, 60 µs lag time, and 500 µs integration time) and the TR-FRET signal was calculated by the ratio of 665 nm/620 nm fluorescent signal. For the fragment screen, on seven of the library plates there were 32-wells of DMSO control, while there were 8-wells of DMSO control in the last plate. The TR-FRET assays for the interactions between His_6_-TTD-PHD and His_6_-UHRF1-FL binding to biotinylated-H3K9me3 (1–20) peptide were similar to the assay for His_6_-TTD. The only difference was that the final concentration of His_6_-TTD-PHD was 200 nM, biotin-H3K9me3 peptide was 200 nM for the His_6_-TTD-PHD TR-FRET assay, while the final concentration of His_6_-UHRF1-FL was 200 nM and biotin-H3K9me3 peptide was 100 nM for the His_6_-UHRF1-FL TR-FRET assay. The buffer pH was 7.5 for the His_6_-TTD-PHD and His_6_-UHRF1-FL assays.

### Protein NMR for characterizing the binding effect of 2,4-lutidine

The BL21(DE3) *E. coli* cells containing pMCSG7-TTD (codon optimized) vector was inoculated into 5 ml ^15^N minimal media (0.1% ^15^NH_4_Cl, 0.5% glucose, 0.1 mM CaCl_2_, 1 mM MgSO_4_, 0.0002% thiamine, 100 µg/ml carboxicillin, 1 × M9 salts, pH = 7.4) and grown at 37 °C until OD_600_ = 0.9. Next, the 5 ml starter culture was added to pre-warmed ^15^N minimal media and grown at 37 °C with shaking until OD_600_ =  ~ 1. After cooling down to 25 °C by 10-min water bath, the expression was induced by 200 µM IPTG at 25 °C overnight. The cell pellet was collected and stored at − 80 °C until use. The ^15^N-labeled TTD was purified by BioRad Nuvia IMAC (Ni) column, TEV protease cleavage followed with another IMAC column clean-up, and finally with Superdex 75 column (22.2 mM Na_2_HPO_4_, pH 7, 2.2 mM DTT, 277.8 mM NaCl). HSQC spectra were acquired at 800 MHz on a Bruker Avance III HD system equipped with a cryoprobe using 256 t1 increments and sweep widths of 12,820.5 Hz (^1^H) and 3243.4 Hz (^15^N). Data were processed and peak-picked in TopSpin 3.6.1. At some 2,4-lutidine concentrations, line broadening hampered accurate peak picking for some resonances; these data points were omitted. Binding curves were fit in Excel to a global K_d_ while allowing the maximum Δδ for each observed resonance to vary independently. Protein and ligand dilution were treated explicitly; samples were prepared at 450 µl, and titration increased the volume by up to 30 µl.

### Protein crystallization and protein: compound co-crystallization

The un-tagged TTD was purified by BioRad Nuvia IMAC (Ni) column, TEV protease cleavage followed with another IMAC column clean-up, and finally with Superdex 75 column. The apo-TTD was crystallized at 0.1 M Bis–Tris, pH = 5.5, 25% w/v PEG 3350 at room temperature. The co-crystals of TTD with 2,4-lutidine were grown in 50 mM Bis–Tris, pH 5.75, 20 mM 2,4-Lutidine, 35% w/v PEG 3350 at room temperature. The crystals were cryo-preserved by 25% glucose and shipped to the Advanced Light Source (Berkeley, CA) for X-ray diffraction. Crystal data were processed using iMOSFLM and the CCP4 suite^70,71^. CC_1/2_ values were used to set resolution cut-offs and assess overall data quality (Supplementary Table S2). Structural refinements were performed using PHENIX, PHASER-MR and COOT^72–74^, phasing was done by molecular replacement using PDB ID 4QQD as a search model. The apo-TTD and TTD/2,4-lutidine co-crystal structures where deposited in the RCSB, PDB IDs: 6W92 and 6VYJ, respectively. Note that crystal soaking was also examined: apo-crystals of UHRF1-TTD were soaked with 2,4-lutidine yet only apo-crystals could be obtained despite multiple efforts. Bis–tris was essential for apo-TTD to crystallize and our apo-TTD structure indicated that bis–tris binds to two sites on TTD. One of the 2,4-lutidine binding sites on TTD in the solved co-crystal structure, the Arg-binding cavity, also binds to bis–tris on apo-TTD. Therefore, it is likely that the failure of TTD crystal soaking by 2,4-lutidine was due to bis–tris occupancy and competition for binding.

### Intrinsic protein fluorescence to characterize 2,4-lutidine binding effect

The untagged TTD was diluted to 30 µM by buffer (50 mM sodium phosphate, pH 7.5, 150 mM NaCl) with or without 60 µM of H3K9me3 peptide (1–20) (Anaspec). 10  µL of diluted TTD was added into 384-well flat bottom black plate (Greiner, Cat# 781086). For the experiment, 2,4-lutidine was directly added to the buffer to make the 20 mM stock for serial dilution (pH is adjusted to 7.5 as 2,4-lutidine is a weak base) and 10  µL of diluted 2,4-lutidine was added to the plate. The final concentration of TTD was 15 µM and the H3K9me3 peptide (if included) was 30 µM. The experiment was performed at room temperature. The 2,4-lutidine effect on the intrinsic fluorescence control protein AviTag-USP7-UBL1/2 (cloning, expression, and purification see supplemental information) was characterized at 3.2 µM protein concentration. After compound addition, the intrinsic tryptophan fluorescence was analyzed by the Tecan Infinite M1000 plate reader (excitation 280 nm, emission 334 nm). The binding of 2,4-lutidine to His_6_-TTD-PHD and C-FLAG-UHRF1 was characterized by similar assay as for TTD, only that the final concentration of protein used was 3.2 µM for His_6_-TTD-PHD and 5 µM for C-FLAG-UHRF1-FL.

### PKA treatment of His-TTD and His-TTD-PHD

The His_6_-TTD and His_6_-TTD-PHD proteins (30 µM) were treated with PKA (P2645, Sigma) at 0.5 U/µl at 30 °C for 1 h in the reaction buffer (50 mM Tris, pH 7.5, 10 mM MgCl_2_, 0.02% Triton-X-100, 1 mM ATP, 1 mM DTT). The untreated sample was incubated at 30 °C for 1 h in same buffer condition without the addition of PKA. After PKA treatment, the protein was used for TR-FRET assay as described above. For identification of phosphorylation site(s) on by LC–MS/MS, His_6_-TTD-PHD (176.7 uM) was treated with PKA at 2U/µl at 30 °C for 1.5 h in the reaction buffer (50 mM Tris, pH 7.5, 10 mM MgCl_2_, 1 mM ATP, 1 mM DTT) with a total volume of 200 µL. For LC–MS/MS, the untreated His_6_-TTD-PHD was incubated at 30 °C for 1.5 h in same buffer condition without the addition of PKA. The PKA-treated and non-treated His_6_-TTD-PHD for LC–MS/MS were tested in same TR-FRET assay as previously described and despite the different reaction condition, similar higher sensitivity to 2,4-lutidine inhibition was observed for PKA-treated His_6_-TTD-PHD (Supplemental Fig. S9).

### Identification of phosphorylated sites on His-TTD-PHD by mass spectrometry

Purified His_6_-TTD-PHD from sham control (no PKA treatment) and PKA treated samples were digested with trypsin at pH 7 (adjusted with ammonium bicarbonate), desalted with 3 M Empore C8 Solid Phase Extraction Disks, dried with a SpeedVac, and resuspended with 2% acetonitrile and 0.1% formic acid, resulting in a final concentration of approximately 1 mM. 1 µL of peptide digest was loaded onto a 10 cm, 100 µm inner diameter C18 column (Waters XBridge C18 3.5 µm 130 Å) self-packed into fused silica pulled to form a nanoelectrospray emitter. Online high-performance liquid chromatography (HPLC) was performed on a Dionex U-3000 Pro-flow system. A 20-min gradient, using buffer A: 2% acetonitrile, 0.1% formic acid and B: 98% acetonitrile and 0.1% formic acid, was used from 0% B to 35% B. The column eluant was introduced into a Thermo Scientific Orbitrap Fusion Lumos by electrospray ionization. A static spray voltage of 2000 V and an ion transfer tube temperature of 320 °C were set for the source. MS1 was performed by the orbitrap (60 k resolution setting) in positive mode with quadrupole isolation. An AGC target of 5.0e5 with 200 ms maximum injection time, one microscan, and a scan range of 500–800 m/z were used. Target precursors 578.2622 m/z ([M + 1] ion for SGPSCK) and 658.2272 m/z ([M + 1] ion for phospho-SGPSCK) were selected for MS2 identification. HCD with stepped collision energy of 15–30–45% was used. MS2 acquisition was performed by orbitrap with 15,000 resolution setting, an AGC target of 1e5, a max injection time of 50 ms, a scan range of 150–2000 m/z, and 2 microscans. Fragments were manually analyzed and matched with a 5 ppm error tolerance, confirming peptide identity. The intensity of the MS1 intact mass signal averaged over the elution window of both peptides was used to detect PKA-mediated kinase activity.

## Supplementary Information

Supplementary Information 1.

## Data Availability

The PDB ID of the apo-TTD structure is 6W92 and the PDB ID of the TTD/2,4-lutidine co-crystal structure is 6VYJ.
