# Supplementary material for: Discovery of small molecules targeting the tandem tudor domain of the epigenetic factor UHRF1 using fragment-based ligand discovery
Source: Sci Rep. 2021 Jan 13;11:1121. doi: 10.1038/s41598-020-80588-4 (PMC7806715; doi:10.1038/s41598-020-80588-4)
Supplement: Supplementary file 1 — Supplementary Information 1. [file 41598_2020_80588_MOESM1_ESM.pdf]

Supporting Information for

Discovery of Small Molecules Targeting the Tandem Tudor Domain of the Epigenetic Factor UHRF1 using Fragment-based Ligand Discovery

Lyra Chang[1,2], James Campbell[1,2], Idris O. Raji[1,2], Shiva K. R. Guduru[1,2], Prasanna Kandel[1,2], Michelle Nguyen[3], Steven Liu[3], Kevin Tran[1], Navneet Krishna Venugopal[1], Bethany C. Taylor[4] ,Matthew V. Holt[4], Nicolas L. Young[4], Errol L. G. Samuel[1,2], Prashi Jain[1,2], Conrad Santini[1,2,5], Banumathi Sankaran[6], Kevin R. MacKenzie[1,2,5], and Damian W. Young[#*1,2,5,7]

[1] Department of Pharmacology and Chemical Biology, Baylor College of Medicine, Houston, TX 77030, USA
[2] Center for Drug Discovery, Baylor College of Medicine, Houston, TX 77030, USA
[3] Department of Chemistry, Rice University, Houston, TX 77030, USA
[4] Department of Biochemistry and Molecular Biology, Baylor College of Medicine, Houston, TX 77030, USA
[5] Department of Pathology and Immunology, Baylor College of Medicine, Houston, TX 77030, USA
[6] Lawrence Berkeley National Laboratory, Berkeley, CA 94720, USA
[7] Therapeutic Innovations Center, Baylor College of Medicine, Houston, TX 77030, USA

[#] Corresponding author

*Correspondence to: Damian.Young@bcm.edu

Table S1. The fragment screen results for two overlapped hits (F1957-0088 and F1957-0227) and F1957-0202, a close homolog for F1957-0088 from Life Chemical Fragment library.

Compound ID	Compound structure	TR-FRET (% change)	AlphaScreen (% change)	ThermoFluor (ΔTm)
F1957-0088	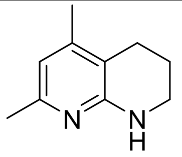	-32.1	-50.7	1.74
F1957-0202	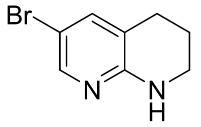	-7.4	-49.1	-0.27
F1957-0227	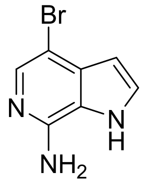	-30	-43	2.58

Table S2. Data collection and refinement statistics

	Apo	2,4-Lutidine
**Data collection**		
Wavelength (Å)		
Space group	P 21 21 21	P 21 21 21
Cell dimensions		
a,b,c (Å)	40.38, 54.87, 66.55	40.28, 55.35, 66.26
α,β,δ (°)	90.00, 90.00, 90.00	90.00, 90.00, 90.00
Resolution (Å)	66.55-1.30	66.26-1.39
R_{sym} or R_{merge}	0.049 (0.153)	0.132 (2.120)
I/σI [a]	20.9 (6.0)	7.0 (0.8)
$CC_{1/2}$ [b]	0.999 (0.967)	0.993 (0.364)
Completeness (%)	97.9 (84.0)	100.0 (99.9)
Redundancy	6.9 (3.5)	
**Refinement**		
Resolution (Å)	42.34-1.30	42.48-1.39
No. reflections	37049	30553
R_{work}/R_{free} [c]	0.174/0.185	0.198/0.226
Ramachandran analysis (% favored/allowed)	98/2	98/2
Average B, all atoms (Å2)	15.0	23.0
R.m.s.[d] deviations		
Bond lengths (Å)	0.008	0.008
Bond angles (°)	0.898	0.984
PDB ID	6W92	6VYJ

[a] Highest resolution shell is shown in parenthesis.
[b] CC1⁄2, Pearson correlation coefficient.
[c] r.m.s., root mean square.
[d] 5.0% of the observed intensities were excluded from refinement for cross validation purposes.

Table S3. Initial filters used for selecting fragments from the Life Chemicals inventory

Property	Criterion
Molecular Weight	< 300
cLogP	< 3
Hydrogen bond acceptors	≤ 6
Hydrogen bond donors	≤ 4
Rotatable bonds	≤ 4
Fraction of sp^3 carbons	< 0.7

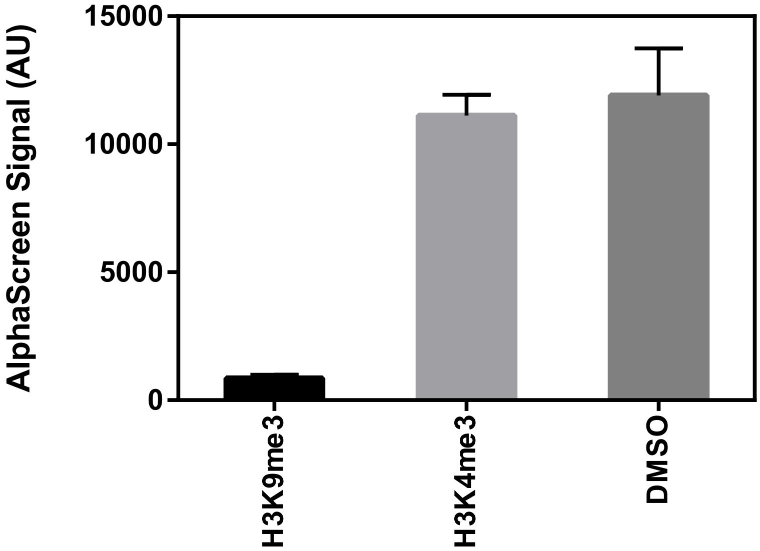

Figure S1. The AlphaScreen signal of N-UHRF1 and C-UHRF1 interaction was specifically inhibited by H3K9me3 (1-20) peptide (50 μM) but not H3K4me3 (1-20) peptide (50 μM). This result suggests that the binding between N-UHRF1 and C-UHRF1 is mostly mediated by TTD-PBR interaction. n=3 for each experimental condition and the error bar represents standard deviation.

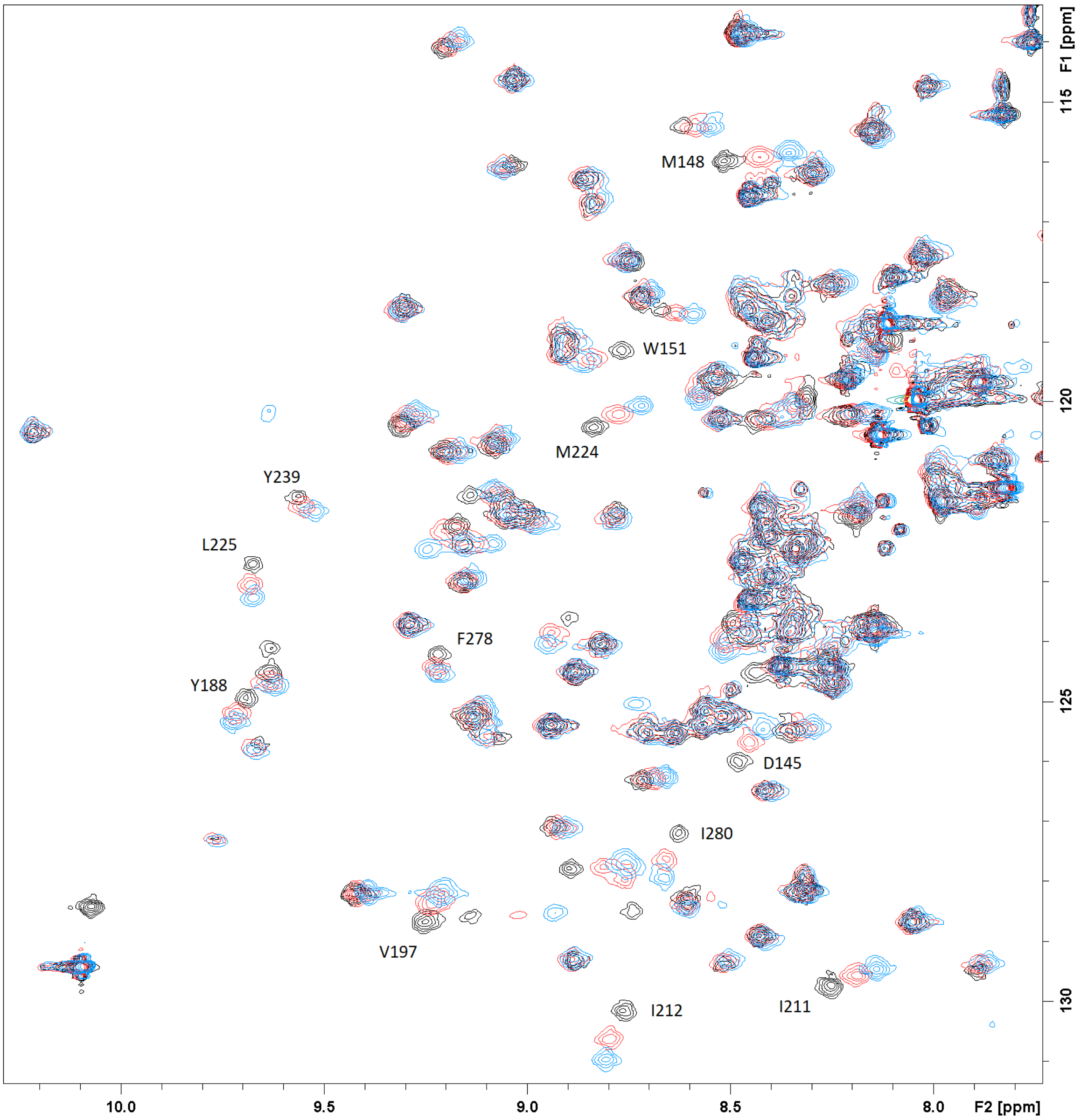

Figure S2. 2,4-lutidine induces chemical shift changes in ^{1}H-^{15}N HSQC spectra of 300 μM TTD. Black, red, and cyan spectra are the same 300 μM TTD sample with 0, 161, and 408 μM 2,4-lutidine added, respectively. Well resolved peaks that undergo chemical shift changes are labeled in the figure close to their apo resonances.

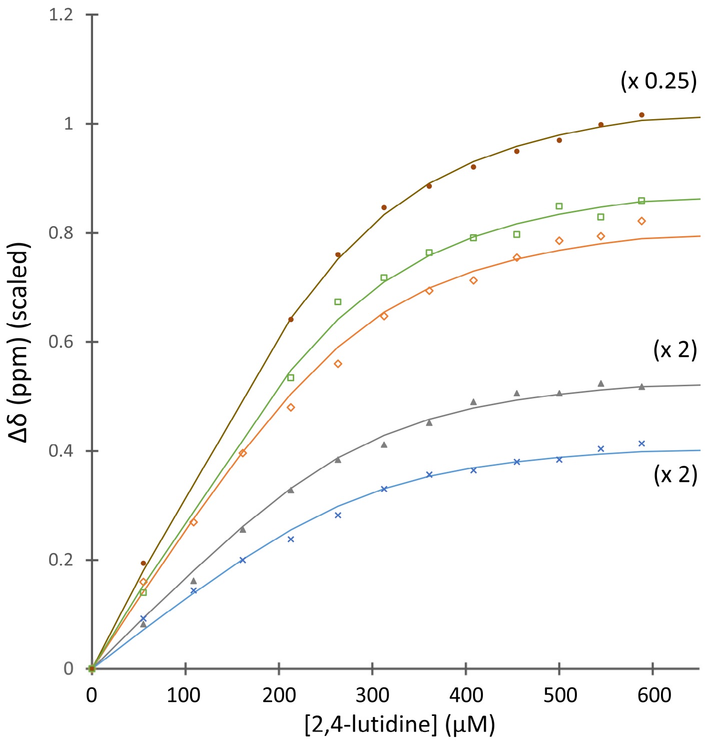

Figure S3. Chemical shift perturbations (Δδ) can be fit to a single binding constant. Chemical shift perturbations of TTD resonances in 2D HSQC spectra upon titration with 2,4-lutidine (Δδ, points) are well predicted by a model (lines) that uses a single Kd (31 μM) and different Δδmax values for each peak. Plotted Δδ values have been scaled as indicated (x 0.25; x 2) to facilitate comparison of the data (points) and best-fit model (lines). Excluding data for the most perturbed peak (solid circles) gives a best-fit Kd of 38 μM.

The model in the fit used to calculate chemical shift changes (Δδcalc) of nucleus *k* is:

$$\Delta\delta calc_k = \Delta\delta max_k \; [\; (P_T + L_T + K_d) - SQRT(\; \{P_T + L_T + K_d\}^2 - 4{*}P_T{*}L_T\;)\;] \;/\; 2{*}P_T$$

where P_T is the total protein concentration (corrected for dilution) and L_T is the total 2,4-lutidine concentration at each experimental titration point, K_d is the dissociation constant, and $\Delta\delta max_k$ is the maximum chemical shift change for nucleus *k*. Best-fit values for K_d and $\Delta\delta max_k$ were obtained by non-linear least squares regression of $\Delta\delta calc_k$ against experimental shift differences ($\Delta\delta obs_k$), using Excel Solver to minimize the sum of squares $(\Delta\delta obs_k - \Delta\delta calc_k)^2$ by varying K_d and all $\Delta\delta max_k$. Values of $\Delta\delta obs_k$ used in the fits were obtained by peak picking referenced 2D spectra in Topspin 3.6.1 and subtracting the chemical shift obtained at the known concentration of lutidine from the chemical shift for the apo protein. Values of the experimental chemical shift changes $\Delta\delta obs_k$ are NOT scaled before fitting, though they are scaled above for display. All lines are calculated using the same K_d value and the $\Delta\delta max_k$ value for each individual resonance.

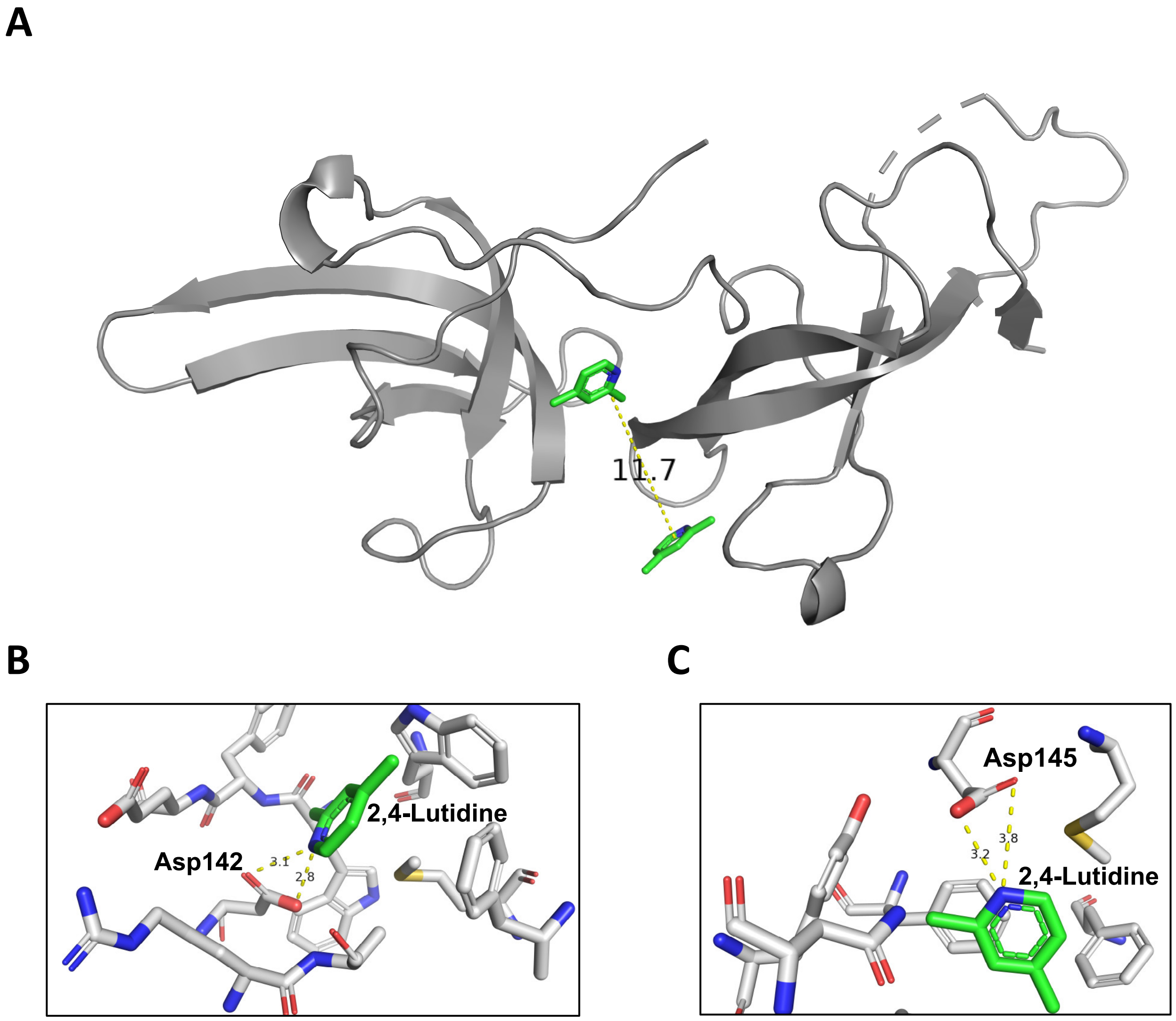

Figure S4. Co-crystal structure of 2,4-Lutidine binding to TTD highlights key molecular interaction. (A) The distance between two 2,4-lutidine is 11.7 Å. (B) In Arg-binding cavity, the distance between the nitrogen of 2,4-lutidine and the carboxylate group of Asp142 is 2.8-3.1 Å. Therefore, 2,4-lutidine and Asp142 likely form a hydrogen bond or salt bridge that stabilizes the fragment:TTD interaction (B) In the aromatic cage, the distance between the nitrogen of 2,4-lutidine and the carboxylate group of Asp145 is 3.2-3.8 Å. Thus, the two may form a weak hydrogen bond or salt bridge that stabilize the interaction.

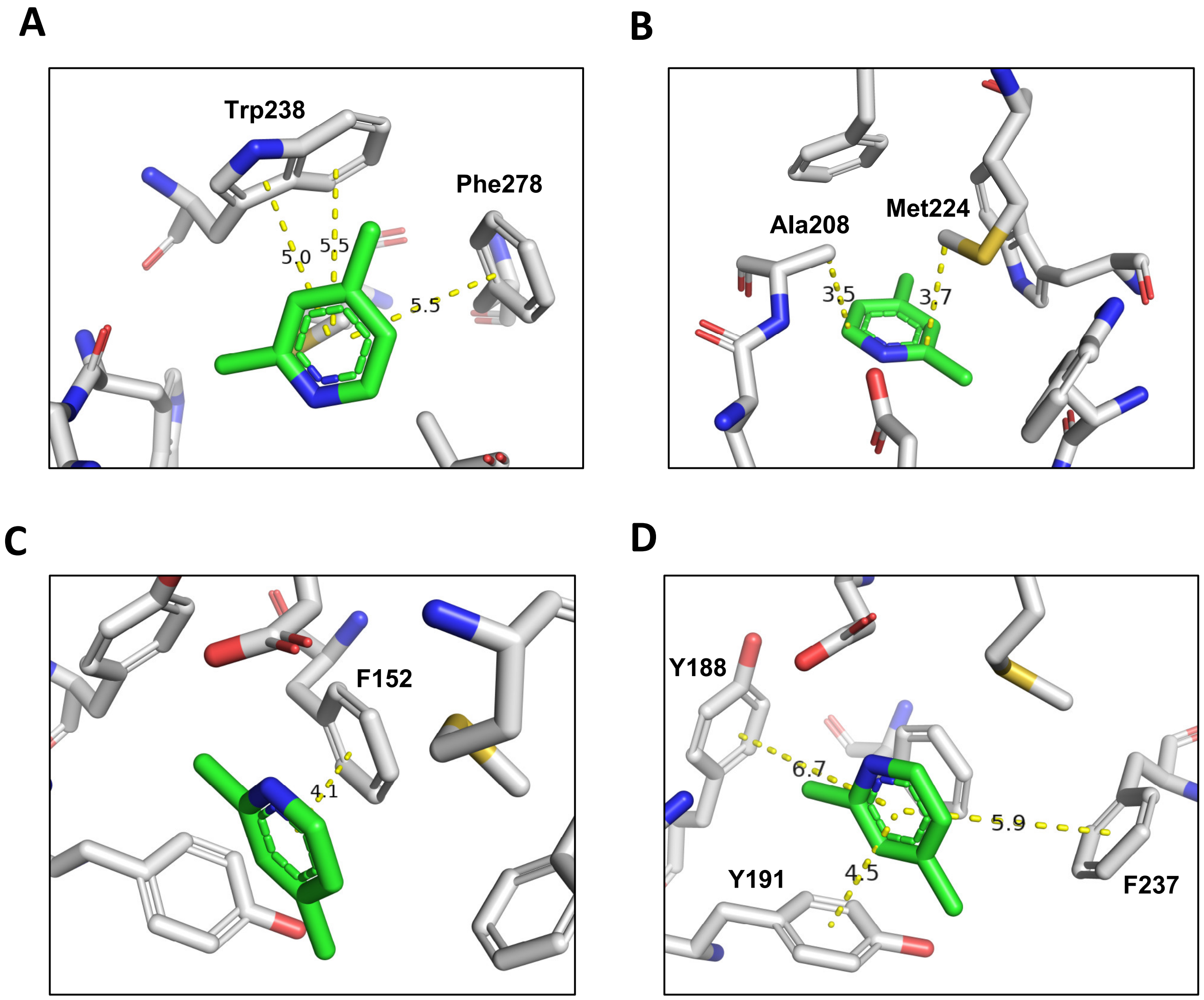

Figure S5. 2,4-lutidine forms hydrophobic interactions with the two binding pockets on TTD. (A) In the Arg-binding cavity of TTD, 2,4-lutidine favorably interacts with Trp238 and Phe278 through π-π T-shaped interaction and (B) makes π-alkyl hydrophobic interactions with Ala208 and Met224. (C) In the aromatic cage of TTD, 2,4-lutidine interacts with Phe152 through π-π stacking while (D) contacting Tyr191 and Phe237 via π-π T-shaped interaction. Note: the numbers on the yellow dotted lines are distances in Å.

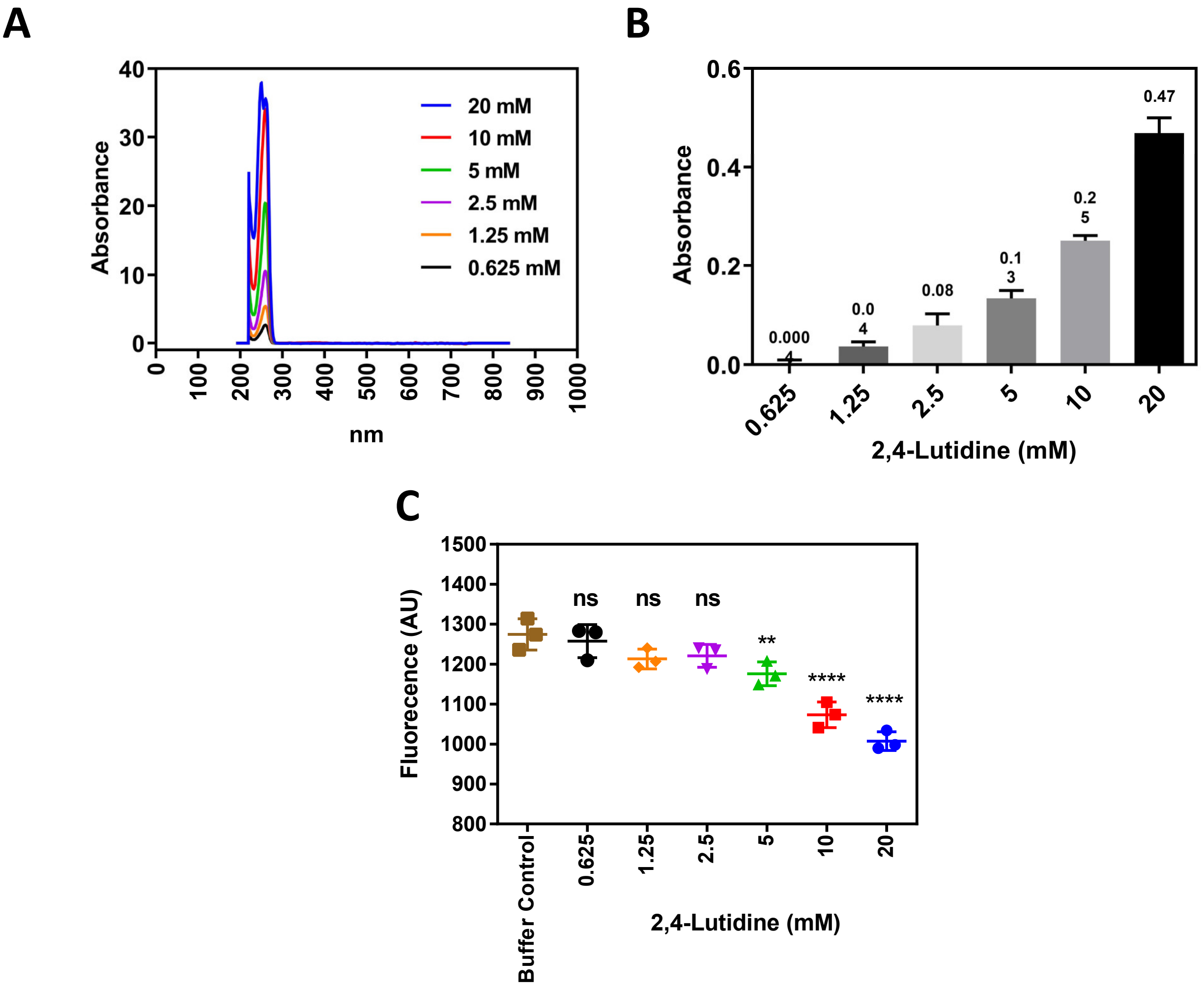

Figure S6. The absorbance and fluorescence profiles of 2,4-lutidine. (A) The absorbance spectra of 2,4-lutidine at different concentrations. (B) The absorbance of 2,4-lutidine of 280 nm light at different concentrations. The absorbance spectra and absorbance of 280 nm light were measured by a Denovix DS-11 spectrophotometer. Each bar is labeled with the average absorbance value from three measurements. (C) concentration-dependent fluorescence of 2,4-lutidine (ex. 280 nm, em. 334 nm). In this experiment, the buffer control had background fluorescence coming from the plastic of the 384-well flat bottom black plate we used. Higher 2,4-lutidine concentrations did not increase 334 nm fluorescence intensity compared to buffer control wells. Therefore, 2,4-lutidine does not emit fluorescence at 334 nm when excited with a 280 nm light. However, at higher 2,4-lutidine concentration (5, 10, and 20 mM), the background fluorescence emission decreased, suggesting that 2,4-lutidine can absorb the 280 nm excitation light. Based on this result, we selected 2.5 mM 2,4-lutidine as the maximum concentration to use in the tryptophan fluorescence experiments to minimize any interference to the result. The data was analyzed using One-way ANOVA analysis (GraphPad Prism6) by comparing each data set with the buffer control.

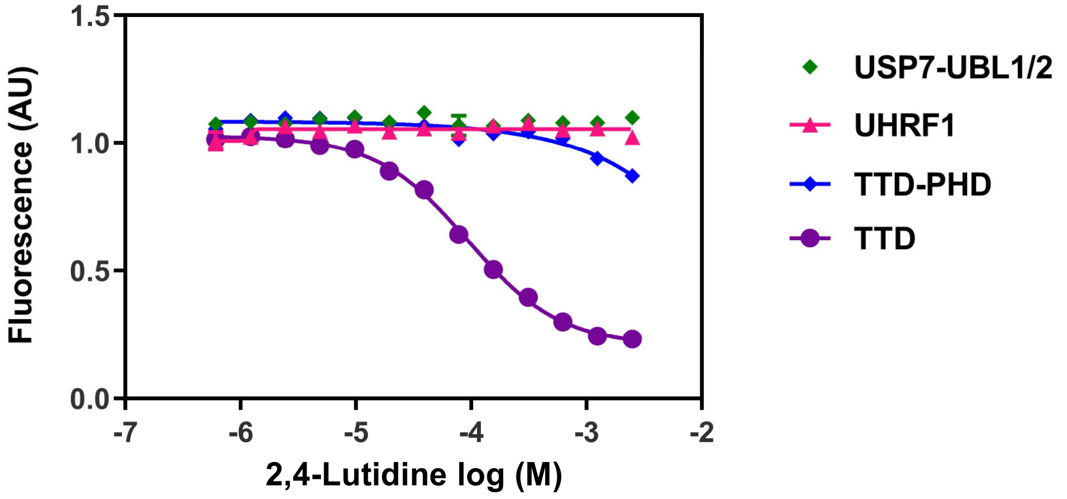

Figure S7. Tryptophan fluorescence monitors 2,4-lutidine binding to His_6-TTD, His_6-TTD-PHD, and C-FLAG-UHRF1-FL. 2,4-Lutidine dose-dependently quenched the tryptophan fluorescence of TTD (purple circle), while only started to show quenching effect against His_6-TTD-PHD at mM concentration (blue diamond). 2,4-Lutidine had no effect on the fluorescence of C-FLAG-UHRF1-FL and the control USP7-UBL1/2 protein. n=3 for each data point. (Note: the standard deviation error bars are included, but the error values are often extremely small in this assay).

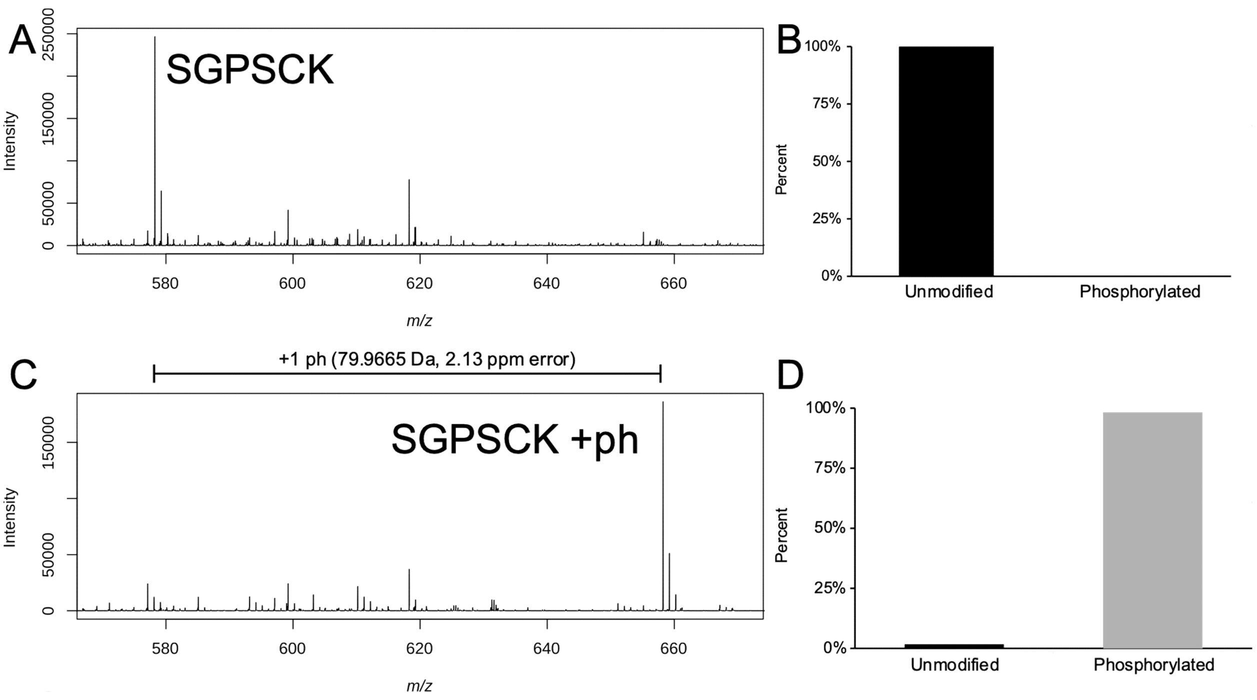

Figure S8. MS analysis of His-TTD-PHD (UHRF1), incubated with and without PKA, shows a dramatic increase in phosphorylation with PKA treatment. (A) Average MS1 spectra of untreated control sample shows no phosphorylation of the targeted peptide. There is a peak at 578.2622 *m/z*, consistent with unphosphorylated target peptide, and no peak at 658.2272 *m/z*. (B) Quantitating the relative amounts of phosphorylated and unphosphorylated peptide for untreated control, 100% is unphosphorylated. (C) Average MS1 spectra of peptide with PKA incubation shows primarily phosphorylated peptide. There is a large peak at 658.2272 *m/z*, consistent with a high abundance of phosphorylated peptide, and a very small peak at 578.2622 *m/z*, consistent with a very low abundance of unphosphorylated peptide. (D) Quantification of the relative amounts of phosphorylated and unphosphorylated peptide in the treatment group (with PKA) shows a much higher abundance of phosphorylated peptide than unphosphorylated peptide (98% relatively phosphorylated). Thus, treatment with PKA mono-phosphorylates this peptide sequence within the intact His-TTD-PHD (UHRF1) substrate.

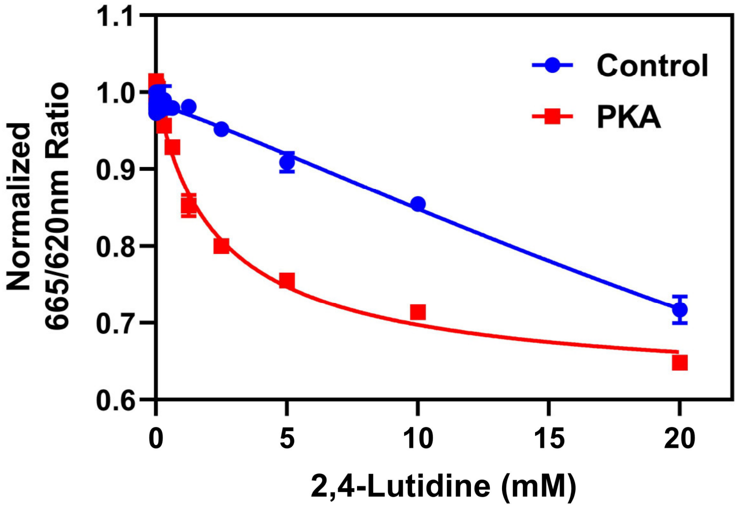

Figure S9. The PKA-treated His_6-TTD-PHD sample used for LC-MS/MS experiment was more sensitive to 2,4-lutidine inhibition of its interaction with H3K3me3 peptide in TR-FRET assay comparing to the non-treated control.
